# Reactive astrocytes facilitate vascular repair and remodeling after stroke

**DOI:** 10.1016/j.celrep.2021.109048

**Published:** 2021-04-27

**Authors:** Michael R. Williamson, Cathleen Joy A. Fuertes, Andrew K. Dunn, Michael R. Drew, Theresa A. Jones

**Affiliations:** 1 Institute for Neuroscience, University of Texas at Austin, Austin, TX 78712, USA; 2 Department of Psychology, University of Texas at Austin, Austin, TX 78712, USA; 3 Department of Biomedical Engineering, University of Texas at Austin, Austin, TX 78712, USA; 4 Center for Learning and Memory, University of Texas at Austin, Austin, TX 78712, USA; 5 Department of Neuroscience, University of Texas at Austin, Austin, TX 78712, USA; 6 Lead contact

## Abstract

Brain injury causes astrocytes to assume a reactive state that is essential for early tissue protection, but how reactive astrocytes affect later reparative processes is incompletely understood. In this study, we show that reactive astrocytes are crucial for vascular repair and remodeling after ischemic stroke in mice. Analysis of astrocytic gene expression data reveals substantial activation of transcriptional programs related to vascular remodeling after stroke. *In vivo* two-photon imaging provides evidence of astrocytes contacting newly formed vessels in cortex surrounding photothrombotic infarcts. Chemogenetic ablation of a subset of reactive astrocytes after stroke dramatically impairs vascular and extracellular matrix remodeling. This disruption of vascular repair is accompanied by prolonged blood flow deficits, exacerbated vascular permeability, ongoing cell death, and worsened motor recovery. In contrast, vascular structure in the non-ischemic brain is unaffected by focal astrocyte ablation. These findings position reactive astrocytes as critical cellular mediators of functionally important vascular remodeling during neural repair.

## In brief

Williamson et al. identify reactive astrocytes as critical mediators of vascular repair and remodeling after stroke. Contrary to the view that reactive astrocytes may limit reparative processes, they show that reactive astrocytes can facilitate at least some aspects of neurovascular repair and behavioral recovery in the injured brain.

## Graphical abstract

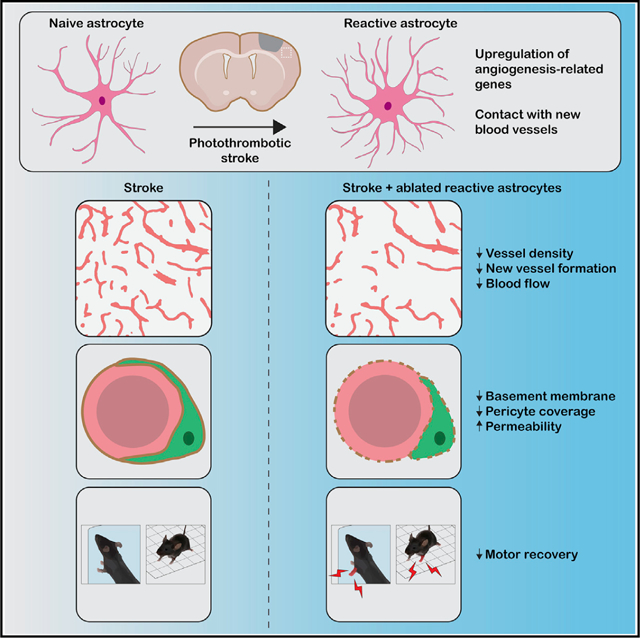

## INTRODUCTION

Reorganization of residual neural tissue underlies recovery after brain injury. Stroke triggers morphological and functional changes across cell types, especially near the infarct (Allegra Mascaro et al., 2019; Brown et al., 2007; Kim et al., 2018). These reparative processes include plasticity of vascular structure and repair of the neurovascular unit (Freitas-Andrade et al., 2020). Vascular remodeling in peri-infarct cortex occurs largely during the first 2 weeks post-stroke in rodent models, and it is characterized by concurrent formation and pruning of capillary segments to help restore blood flow to residual tissue (Williamson et al., 2020). This process contributes to behavioral recovery and is thought to be crucial for resolving metabolic failure in surviving tissue and re-establishing a microenvironment that can support other aspects of neural repair (Mostany et al., 2010; Rust et al., 2019a; Williamson et al., 2020). However, the factors that mediate vascular remodeling after cerebral ischemia are incompletely understood.

Astrocytes interact closely with blood vessels through endfeet, which normally completely cover cerebral microvessels (Mathiisen et al., 2010). During developmental angiogenesis, astrocytes aid vascular development and maturation by providing a physical scaffold and producing pro-angiogenic factors (Reemst et al., 2016). Astrocytes have crucial roles in mediating neurovascular coupling, in which local blood flow is regulated to match the energy demands of neural activity (Mishra et al., 2016; Nortley and Attwell, 2017). Neurovascular coupling is impaired after stroke (Attwell et al., 2010; He et al., 2020), and this is at least in part due to astrocytic dysfunction (Howarth et al., 2017). Astrocytes have been implicated in homeostatic maintenance and post-injury repair of the blood-brain barrier (Bush et al., 1999; Faulkner et al., 2004; Heithoff et al., 2021; Vivinetto et al., 2020). For example, acute astrocyte death was reported to cause leakage of small and large blood-borne molecules into brain parenchyma (Heithoff et al., 2021; cf. Schreiner et al., 2015). Moreover, astrocyte ablation increased myeloid cell infiltration and immunoglobulin G extravasation in brain stab wound and spinal cord injury models (Bush et al., 1999; Faulkner et al., 2004). Several types of interactions between astrocytes and vasculature may be relevant in the context of post-stroke recovery.

Injury induces reactive astrogliosis in which astrocytes undergo changes in morphology and function (Burda and Sofroniew, 2014). While it is unclear how reactive astrocytes influence vascular remodeling, reactive astrogliosis has been studied extensively in the context of post-injury axonal plasticity. Reactive astrocytes express factors that inhibit axon growth, which has led to the idea that reactive astrocytes limit spontaneous repair after injury (Bush et al., 1999; Liuzzi and Lasek, 1987; Sofroniew and Vinters, 2010). However, recent work indicates that the expression of growth inhibitory factors in reactive astrocytes is minor relative to other cell types. For example, spinal cord injury increases expression of axon-growth-inhibitory chondroitin sulfate proteoglycans, but this is primarily driven by non-astrocytes (Anderson et al., 2016). Moreover, reactive astrocytes produce an array of growth-promoting factors that allow them to facilitate axonal outgrowth after spinal cord injury (Anderson et al., 2016). Importantly, there are major similarities in the mechanisms underlying axonal and vascular growth (Carmeliet and Tessier-Lavigne, 2005), including morphological similarities of axonal growth cones and endothelial tip cells, as well as overlap in the guidance cues that mediate their outgrowth (Carmeliet and Tessier-Lavigne, 2005; Rust et al., 2019a; Wälchli et al., 2013). Therefore, the principles of facilitation of neuronal outgrowth by reactive astrocytes may be relevant for vascular sprouting.

Based on evidence that (1) astrocytes and endothelial cells interact closely in the neurovascular unit, (2) reactive astrocytes can facilitate and are necessary for axon regeneration, (3) neuronal and vascular sprouting share common mechanisms, and (4) genes involved in vascular repair are upregulated in astrocytes after injury, we hypothesized that reactive astrocytes might protect and repair pre-existing vessels and support new vessel formation after cerebral ischemia. Herein, we report that ablation of a subset of reactive astrocytes surrounding cortical infarcts dramatically increases vessel loss and impairs vascular remodeling and repair of the neurovascular unit. This disruption of vascular repair is accompanied by exacerbated cerebral blood flow deficits and worsened behavioral recovery. These findings position reactive astrocytes as a major regulator of vascular repair and remodeling after cerebral ischemia. Thus, contrary to the view that reactive astrocytes may limit reparative processes, reactive astrocytes have active roles in facilitating at least some aspects of neurovascular repair and behavioral recovery.

## RESULTS

### Stroke activates transcriptional programs relevant for vascular remodeling in reactive astrocytes

**Figure 1.**
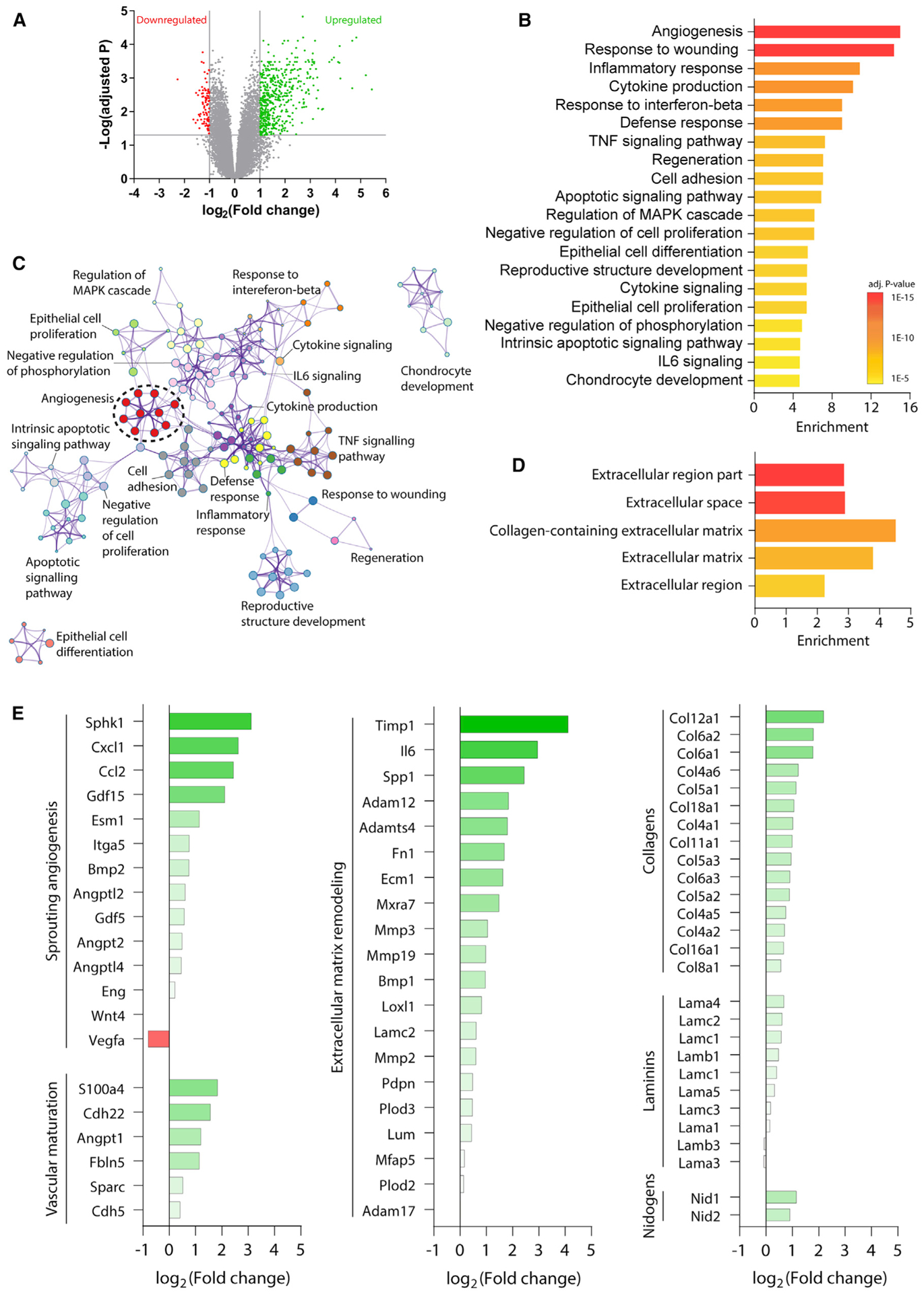
Stroke activates transcriptional programs relevant for vascular remodeling in reactive astrocytes (A) Volcano plot of gene expression in astrocytes after stroke relative to sham procedures. Genes with expression increased log_2_ fold change >1 and adjusted p < 0.05 are shown in green. Genes with expression decreased log_2_ fold change less than −1 and adjusted p < 0.05 are shown in red. (B) Top 20 enriched biological process and function gene ontology term clusters among upregulated genes. Terms are sorted by enrichment and color coded by p value. (C) Enrichment network plot of the top enriched gene ontology term clusters among upregulated genes. Enriched terms are indicated as nodes. Nodes are color coded by cluster membership. Nodes are connected based on gene member similarities. (D) Top five enriched cellular component terms for protein products of upregulated genes in reactive astrocytes. (E) Relative expression of genes associated with intercellular regulation of sprouting angiogenesis, vascular maturation, extracellular matrix remodeling, and extracellular matrix components. Genes associated with intercellular regulation of these processes were defined as those that code for proteins that are secreted, involved in the production of secreted molecules, or located on the cell surface. Data are expressed as log_2_ fold change in astrocytes from stroke versus sham animals.

We analyzed a reactive astrocyte transcriptome resource (GEO: GSE35338) (Zamanian et al., 2012) to elucidate functional changes in reactive astrocytes during the first week after experimental stroke, which is a time of significant vascular plasticity and repair (Williamson et al., 2020). Samples were collected from 30-to 35-day-old Aldh1l1-EGFP mice on a Swiss Webster background subjected to 60-min middle cerebral artery occlusion or sham procedures (Zamanian et al., 2012). Astrocytes were purified by fluorescence-activated cell sorting and gene expression was quantified by microarray. There were 383 upregulated and 70 downregulated genes in reactive astrocytes during the first week after stroke relative to sham-operated controls (Figure 1A). Gene Ontology (GO) analysis of the upregulated genes identified “angiogenesis” as the top enriched biological process (Figures 1B and 1C). In addition, cellular component GO analysis revealed extracellular regions as the top enriched targets for protein products of upregulated genes (Figure 1D). Upregulated genes included those coding for proteins involved in intercellular regulation of sprouting angiogenesis (e.g., *Cxcl1*, *Esm1*, *Sphk1*) and vessel maturation (e.g., *Angpt1*, *Cdh22*, *S100a4*) (Buga et al., 2014; Rust et al., 2019b; Figure 1E; Figure S1). Moreover, many genes classified as implicated in angiogenesis were shared by other top enriched GO terms, making it a central node in the network of enriched processes (Figure 1C). These data indicate that upregulation of genes relating to vascular growth and repair are a central component of the transcriptional programs activated in reactive astrocytes after ischemia.

Blood vessels are encased in a basement membrane composed of extracellular matrix proteins, which are in part produced by astrocytes. Ischemia induces significant remodeling of the basement membrane, which is critical for vascular plasticity and maturation (Davis and Senger, 2005). “Extracellular matrix organization” was another GO term significantly enriched among upregulated transcripts in reactive astrocytes (Figure S2). Upregulated genes implicated in extracellular matrix remodeling included *Ecm1*, *Timp1*, and several matrix metalloproteinases (Figure 1E). Expression of basement membrane components was also increased in astrocytes after stroke, including isoforms of proteins that compose the majority of brain vascular basement membrane (collagen IV α1 and α2; laminin α4, α5, β1, and γ1; and nidogen-1 and −2; Figure 1E; Kang and Yao, 2020). These results suggest that reactive astrocytes undergo gene expression changes in response to stroke that enable them to influence vascular and extracellular matrix remodeling. Taken together, these data led us to investigate whether reactive astrocytes have a functional role in promoting post-stroke vascular remodeling.

### Astrocytes interact with remodeling vessels after stroke

**Figure 2.**
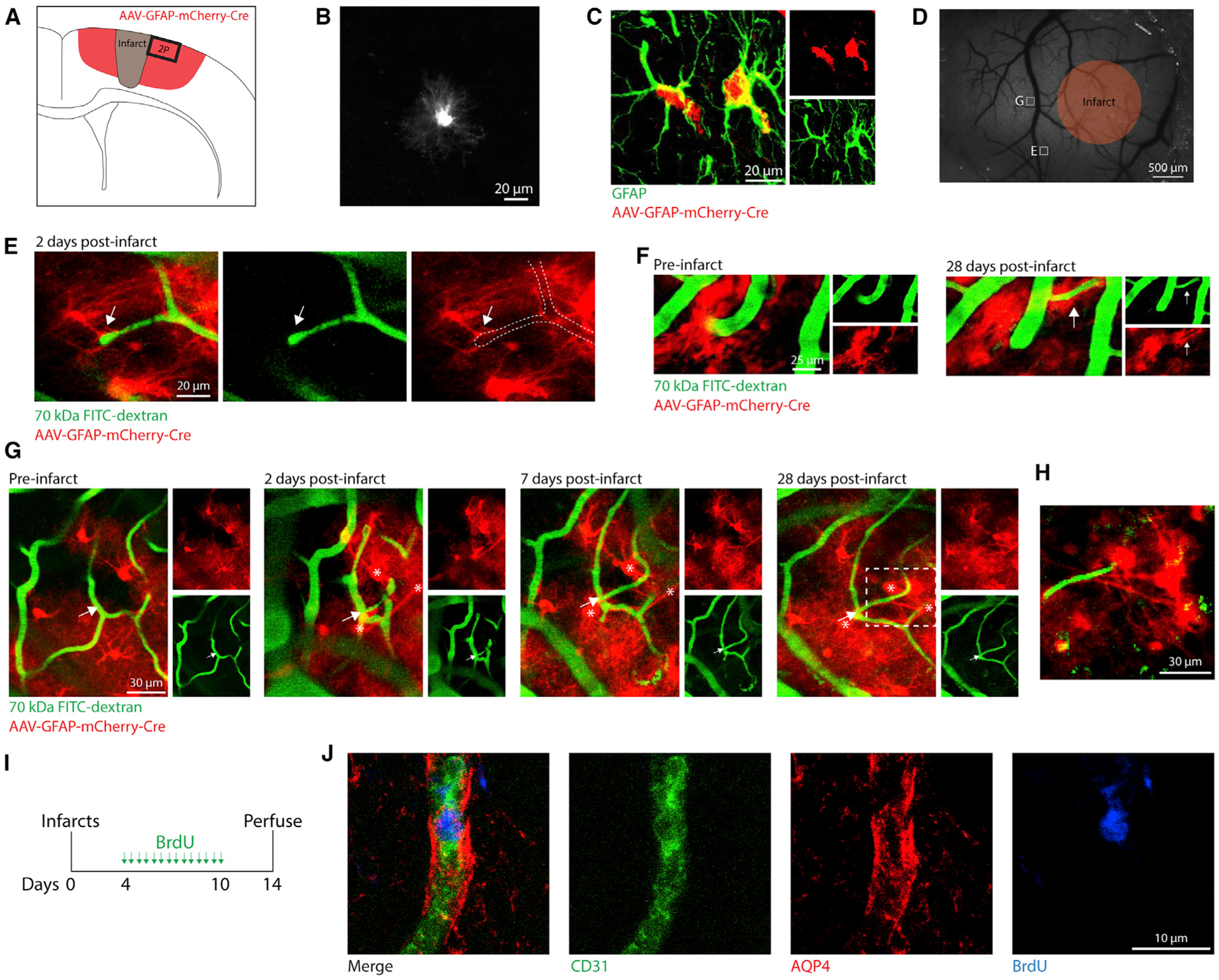
Astrocytes interact with remodeling vessels after stroke (A) Illustration of AAV-mediated labeling and imaging. (B) *In vivo* two-photon (2P) image of an AAV-labeled cell with characteristic astrocyte morphology (pre-stroke). (C) Confocal image validating AAV-mediated labeling of astrocytes with immunohistochemistry. (D) Wide-field speckle contrast image of the brain surface. Orange region represents the approximate boundaries of the infarct core. White boxes indicate 2P-imaged regions corresponding to labeled images. (E) 2P image from 2 days post-stroke of a vascular sprout contacted by a nearby astrocyte (arrow). This region was only imaged at 2 days post-stroke. (F) 2P images showing a vessel that reoriented and increased diameter after stroke ensheathed by astrocytes (arrow). (G) 2P images showing a new capillary segment (arrow) formed after stroke contacted by astrocytes (*). (H) 2P image corresponding to the dashed box in (G) showing astrocytes extending processes that terminate on the new vessel segment. (I) Experimental design for labeling proliferating vessels after stroke. (J) Confocal images showing a new vessel (CD31^+^BrdU^+^) ensheathed by astrocytic endfeet (AQP4^+^). All CD31^+^BrdU^+^ new vessels analyzed (76/76 new vessels from six mice) were surrounded by AQP4^+^ endfeet.

Having identified post-ischemic transcriptional changes in reactive astrocytes related to vascular remodeling, we next examined the interactions of astrocytes with newly formed vessels after photothrombotic infarcts *in vivo*. To sparsely label reactive astrocytes, we injected AAV-GFAP-mCherry-Cre throughout sensorimotor cortex of tdTomato Cre-reporter mice (Ai14; Rosa-CAG-LSL-tdTomato) immediately prior to installation of cranial windows (Figures 2A–2C; Figure S3). This approach produced an incomplete labeling of astrocytes due to a combination of lack of *Gfap* promoter activity in some cells and others not being transfected due to distance from AAV injection sites. Although not all non-reactive astrocytes express substantial GFAP protein, *Gfap* promoters allow for efficacious expression of transgenes in most astrocytes in the presence or absence of injury (Griffin et al., 2019; Hu et al., 2020; Yu et al., 2020). After several weeks of recovery, we two-photon imaged labeled astrocytes and fluorescein isothiocyanate (FITC)-dextran-labeled vessels before and after producing photothrombotic infarcts in motor cortex through the cranial window (Clark et al., 2019a). After infarcts were produced, we observed the formation of a small number of new vessel segments in some peri-infarct regions (~72 new segments per mm^3^ averaged across all imaging locations). At early (2 days) and later (up to 28 days) time points relative to stroke, labeled astrocytes were found adjacent to and contacting or wrapping around new vessel sprouts and segments (Figures 2D–2H). Astrocytes also covered pre-existing vessel segments that underwent substantial changes in orientation and diameter (Figure 2F). Our sparse labeling approach did not allow us to quantify the extent of astrocytic coverage of vessels from our imaging dataset.

To confirm that astrocytic processes interact with newly formed vessels in peri-infarct cortex, we subjected six wild-type mice to photothrombotic infarcts and administered bromodeoxyuridine (BrdU) twice per day from days 4 to 10 post-stroke to label proliferating vessels (Figure 2I). At 14 days post-stroke, we stained for BrdU and CD31, to label new endothelial cells, and aquaporin 4 (AQP4), which is localized to the endothelial aspect of astrocytic endfeet (Haj-Yasein et al., 2011). Of the 76 BrdU^+^CD31^+^ vessels we examined in peri-infarct cortex, all were surrounded by AQP4^+^ endfeet (Figure 2J). Our observations show that astrocytes associate with newly developing vascular segments during post-stroke neovascularization. This active coverage of vessels is consistent with a study in which laser ablation of single astrocytic endfeet resulted in the rapid astrocytic recoverage of exposed vessel segments (Kubotera et al., 2019). Our results provide anatomical evidence for interactions between reactive astrocytes and remodeling vasculature after stroke.

### Chemogenetic ablation of a subset of reactive astrocytes worsens behavioral function after cortical infarcts

**Figure 3.**
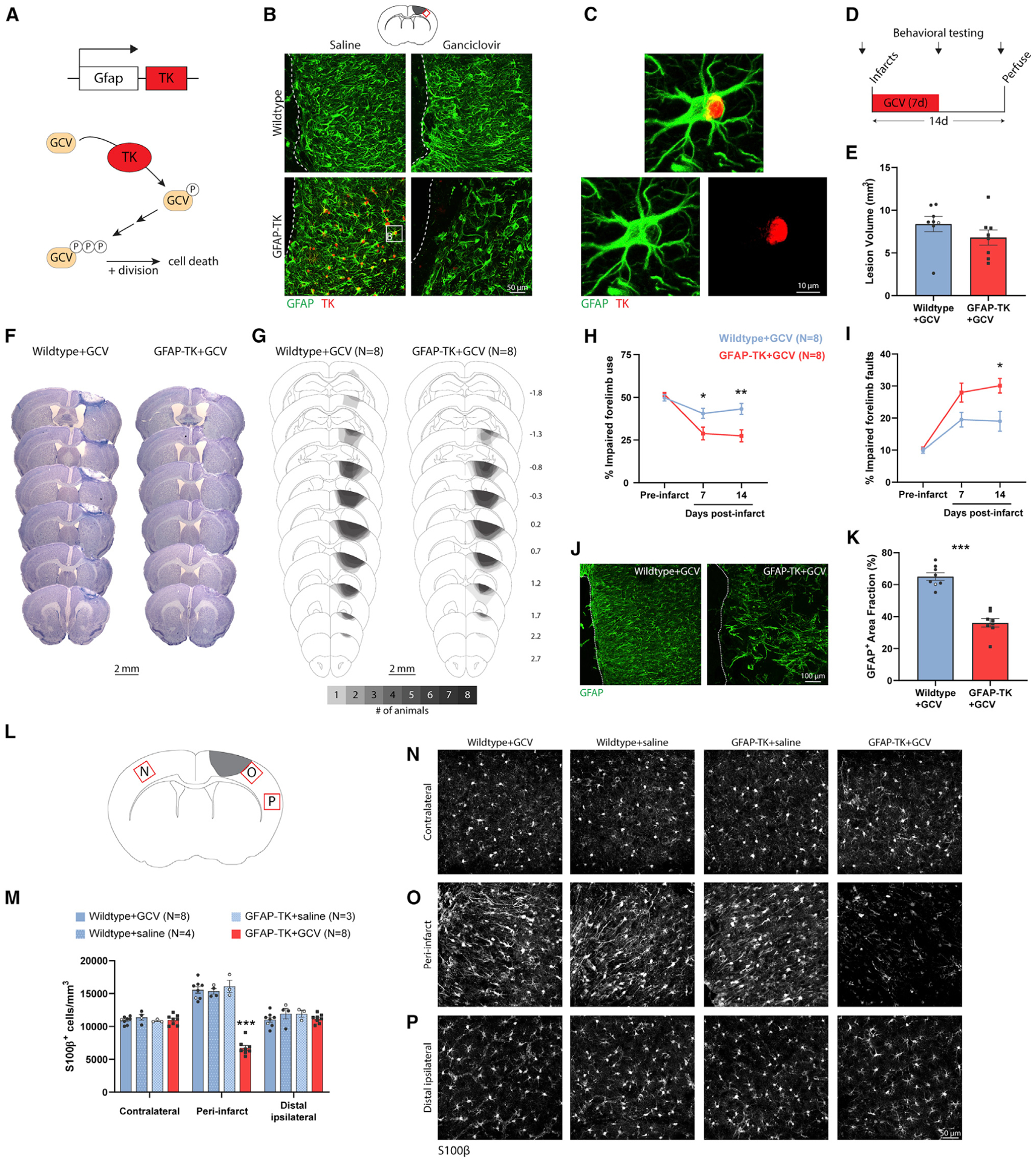
Chemogenetic ablation of a subset of reactive astrocytes worsens behavioral function after cortical infarcts (A) Schematic illustrating how *Gfap* promoter-driven expression of thymidine kinase (TK) permits ganciclovir (GCV)-conditional ablation of dividing cells. (B) Confocal images from peri-infarct cortex illustrating conditional ablation of a subset of astrocytes in peri-infarct cortex specific to GFAP-TK mice given GCV. Dashed lines indicate the lesion border. The sampling region is indicated by the red box in the diagram at top. Images are from 10 days post-stroke. GCV or vehicle (saline) was delivered via osmotic pump for the first 7 days post-stroke. White box in the lower left image corresponds to the image in (C). (C) High-magnification image of a GFAP^+^TK^+^ astrocyte in a GFAP-TK mouse given saline. (D) Experimental timeline. n = 8 mice/group (wild-type versus GFAP-TK). (E) Lesion volume measured at 14 days was not different between groups (t_(14)_ = 1.27, p = 0.225). (F) Representative Nissl-stained coronal sections from 14 days post-stroke. (G) Schematic lesion reconstruction. Numbers on right indicate distance (mm) relative to bregma. (H and I) Astrocyte ablation worsened motor function as measured with the cylinder (H) and grid walking tests (I). *p < 0.05, **p < 0.01, Holm-Sidak’s tests comparing groups at each time point. (J and K) Confocal images (J) and quantification (K) of peri-infarct GFAP expression, confirming ablation of a subset of astrocytes in GFAP-TK mice given GCV. ***t_(14)_ = 8.17, p < 0.0001. (L) Schematic showing sampling regions for (N)–(P). (M) Quantification of S100β^+^ astrocyte density by region at 14 days post-stroke. Astrocyte ablation was specific to GFAP-TK+GCV mice. ***p < 0.0001, Tukey tests compared with other groups in the peri-infarct region. (N–P) Confocal images of S100β^+^ astrocytes in contralateral cortex (N), peri-infarct cortex (O), and ipsilateral cortex ~2 mm from the infarct border (P, “distal ipsilateral”). Data are presented as mean ± SEM. When replicates are shown, data points representing males are shown as filled symbols; data points representing females are shown as open symbols.

To directly examine contributions of reactive astrocytes to behavioral recovery and vascular remodeling we used transgenic mice expressing thymidine kinase (TK) under control of the *Gfap* promoter (GFAP-TK) to conditionally ablate a subset of reactive astrocytes after stroke. Administration of high-dose ganciclovir (GCV), a TK substrate, during the first week after CNS injury leads to the ablation of proliferating reactive astrocytes through production of nucleotide analogs that induce apoptosis during cell division (Figure 3A; Anderson et al., 2016; Bush et al., 1999). This system permitted inducible and selective ablation of a subset of reactive astrocytes near the lesion border (Figure 3B; Bush et al., 1999; Faulkner et al., 2004). Wild-type littermates given GCV and TK^+^ mice given vehicle underwent uncompromised astrogliosis (Figures 3B and 3C).

We induced focal ischemia by photothrombosis in the forelimb area of motor cortex in wild-type (n = 8) and GFAP-TK mice (n = 8) (Lee et al., 2004; Tennant et al., 2011). Immediately prior to photothrombosis, mice were implanted with osmotic pumps to deliver GCV during the following week, causing ablation of proliferating astrocytes in GFAP-TK mice. Mice were euthanized 2 weeks post-stroke (Figure 3D). We chose this survival time because most neovascularization occurs during the first 2 weeks post-stroke in this model (Williamson et al., 2020). Lesion volume and location were not different between groups (Figures 3E–3G). Stroke caused significant impairments in forelimb motor function assayed with the cylinder (significant group by time interaction, F_(2,28)_ = 8.1, p = 0.002, two-way repeated-measures ANOVA) and grid walking (significant group by time interaction, F_(2,28)_ = 4.7, p = 0.018, two-way repeated-measures ANOVA) tests. These tests measure forelimb use asymmetry and locomotor coordination, respectively. Astrocyte ablation significantly worsened behavioral performance on both tests relative to wild-type mice with intact astrocytes (Figures 3H and 3I). Astrocyte ablation was confirmed in GFAP-TK mice by a significantly reduced GFAP^+^ area fraction in peri-infarct cortex (Figures 3J and 3K). Stereological counts of S100β^+^ astrocytes confirmed that astrocyte ablation was specific to peri-infarct cortex in GFAP-TK mice given GCV (Figures 3L–3P). These results illustrate the effectiveness and specificity of chemogenetic ablation of reactive astrocytes in GFAP-TK mice and show that ablation of a subset of astrocytes in peri-infarct cortex worsens functional outcome.

### Astrocyte ablation impairs vascular remodeling and exacerbates vascular permeability

**Figure 4.**
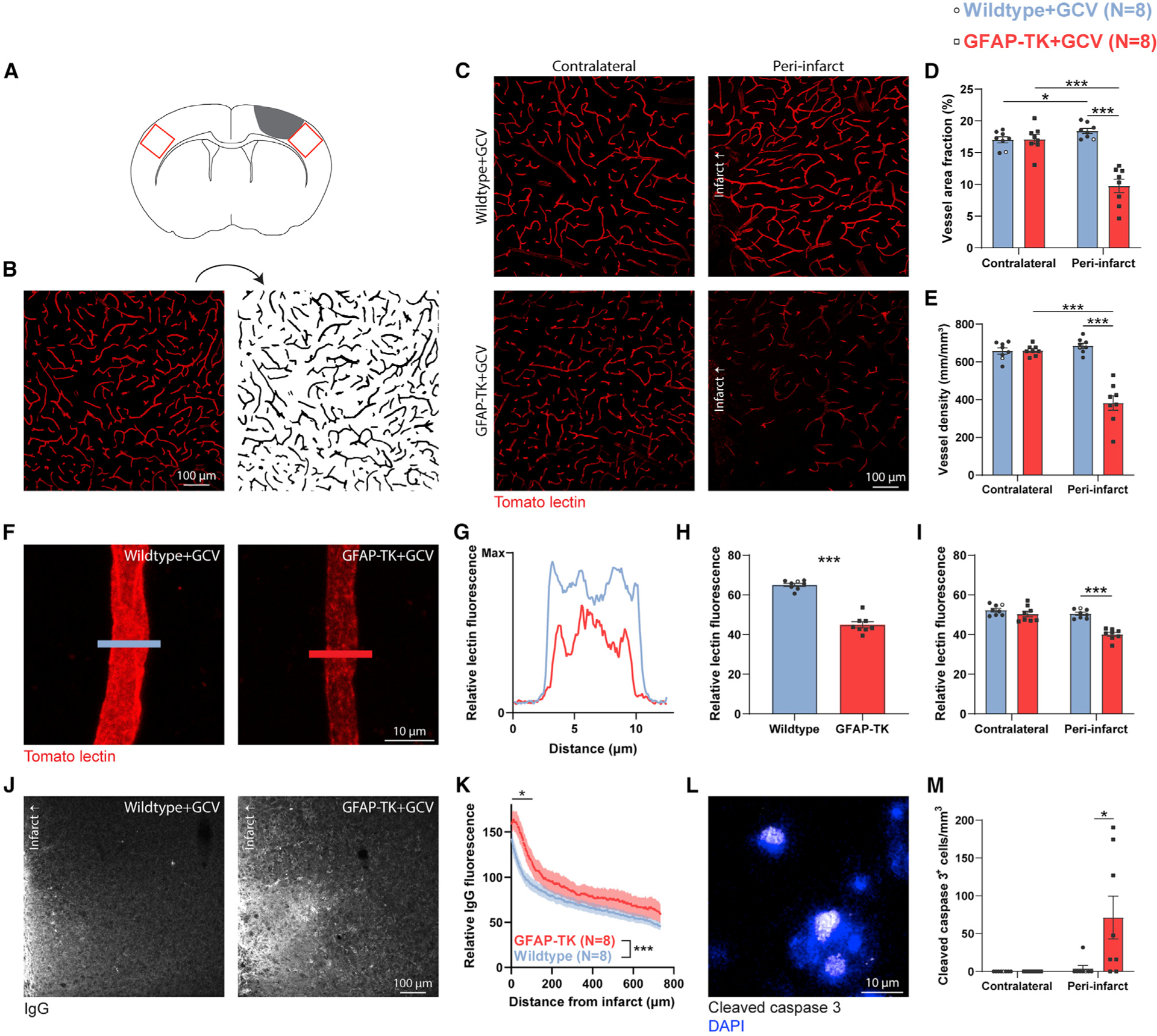
Astrocyte ablation impairs vascular remodeling and exacerbates vascular permeability, which is associated with chronic cell death (A) Sampling regions for images from peri-infarct and homotopic contralateral cortex (red boxes). (B) Example of processing and binarization of a confocal image of tomato lectin-labeled vasculature. (C–E) Confocal images (C) and quantification (D and E) of vasculature from 14 days post-stroke show that astrocyte ablation markedly impaired vascular remodeling in peri-infarct cortex, as measured by reduced vascular area fraction and length. *p < 0.05, ***p < 0.001, t tests (n = 8 mice/group). (F) Representative high-magnification images of lectin-labeled vessels. (G) Lectin fluorescence intensity measured across line profiles as indicated in (F). (H and I) Quantification of glycocalyx coverage as lectin fluorescence from line profiles (H) and within vessel masks (I). ***t_(14)_ ≥ 8.1, p < 0.0001. (J and K) Representative confocal images (J) and quantification (K) of IgG demonstrate increased vascular permeability in peri-infarct cortex of GFAP-TK mice. Two-way ANOVA showed significant effects of distance (F_(484,6790)_ = 11.6, p < 0.0001) and group (F_(1, 6790)_ = 836.3, p < 0.0001). Comparison between mean fluorescence in 50-μm bins showed a significant difference between groups within 100 μm from the infarct border (*t_(14)_ ≥ 2.2, p ≤ 0.043, t tests). (L and M) Confocal image (L) and quantification (M) of cleaved caspase-3^+^ cells. Astrocyte ablation significantly increased the number of apoptotic cleaved caspase-3^+^ cells in peri-infarct cortex at 14 days post-stroke. *U = 11, p = 0.018, Mann-Whitney U test. No cleaved caspase-3^+^ cells were observed in homotopic contralateral cortex. Data are presented as mean ± SEM. When replicates are shown, data points representing males are shown as filled symbols; data points representing females are shown as open symbols.

Cortical photothrombotic infarcts cause elimination of some pre-existing vessels and formation of new vessels in residual peri-infarct cortex (Williamson et al., 2020). Most of this vascular remodeling takes place during the first 2 weeks post-stroke (Williamson et al., 2020). To examine effects of astrocyte ablation on vascular remodeling we injected mice retro-orbitally with tomato lectin immediately before euthanasia to label vasculature on day 14 post-stroke (Figures 4A–4C; Figure S4). Analysis of vasculature in peri-infarct and homotopic contralateral cortex demonstrated that the vascular area fraction was increased in peri-infarct cortex of wild-type mice relative to intact cortex (Figure 4D), providing evidence of changes in vascular structure after stroke. In contrast, vascular area fraction and length were significantly reduced in peri-infarct cortex of GFAP-TK mice relative to intact contralateral cortex and wild-type peri-infarct cortex (Figures 4D and 4E). Therefore, astrocyte ablation results in substantially reduced peri-infarct vessel density after stroke. This may be attributable to reactive astrocytes preventing vessel loss and/or promoting neovascularization.

We noticed that in addition to sparse vascularization in peri-infarct cortex of mice with ablated astrocytes, fluorescence of existing vessels was often reduced relative to wild-type controls. Tomato lectin binds to the endothelial glycocalyx, which is a layer of glycoproteins along the luminal surface of blood vessels. Several cerebrovascular diseases are associated with glycocalyx loss (Yoon et al., 2020). Glycocalyx is required for developmental neovascularization (Henderson-Toth et al., 2012) and contributes to blood-brain barrier properties (Kutuzov et al., 2018). We used lectin fluorescence to measure glycocalyx coverage with two approaches (Yoon et al., 2020). First, we measured lectin fluorescence in cross-sectional line profiles from peri-infarct capillary segments (Figures 4F and 4G). This approach produced measurements independent of segmented masks (i.e., independent of image partitions based on vascular fluorescence intensity). The mean fluorescence across peri-infarct vessels was significantly reduced in GFAP-TK mice (Figure 4H). Second, we used binarized vessel masks to measure mean fluorescence across all vessels, which provides a more complete assessment of glycocalyx coverage, but necessarily relies on segmented masks. Again, glycocalyx content was reduced in peri-infarct cortex of mice with ablated astrocytes (Figure 4I). Fluorescence in contralateral cortical vessels was not different between groups. Thus, reactive astrocyte loss causes reduced endothelial glycocalyx content in peri-infarct cortex. Future studies could confirm this finding with ultrastructural analysis. Since glycocalyx contributes to the exclusion of large molecules across the blood-brain barrier (Kutuzov et al., 2018), we reasoned that vascular permeability could be increased in GFAP-TK mice.

Stroke causes substantial disruption of the blood-brain barrier, allowing pathological extravasation of blood-borne molecules into brain parenchyma (Freitas-Andrade et al., 2020; Munji et al., 2019). Effective vascular repair involves reestablishment of barrier function (Rust et al., 2019a, 2019c). To examine whether the failure of vascular repair in GFAP-TK mice was coupled with increased vascular permeability, we stained for mouse immunoglobulin G (IgG), which is usually excluded from brain parenchyma (Munji et al., 2019). IgG fluorescence was markedly increased near the infarct border and declined with distance into residual cortex (Figures 4J and 4K). IgG leakage was exacerbated in mice with ablated astrocytes, indicating greater vascular permeability. This is consistent with findings of greater blood-brain barrier dysfunction after manipulations to attenuate astrogliosis in other injury models (Bush et al., 1999; Faulkner et al., 2004; Vivinetto et al., 2020).

Both sparse vascularization and enhanced vascular permeability in peri-infarct cortex could contribute to cell death (Moeini et al., 2018; Munji et al., 2019; Zlokovic, 2008). We assessed cell death in contralateral and peri-infarct cortex at day 14 by staining for pro-apoptotic proteolytically cleaved caspase-3 (Figure 4L). We did not observe any cleaved caspase-3^+^ cells in contralateral cortex, and only few were found in peri-infarct cortex of wild-type mice, consistent with findings that peri-infarct apoptotic cell death largely resolves within several days after focal ischemia (Broughton et al., 2009). In contrast, there was a nearly 18-fold increase in the number of cleaved caspase-3^+^ cells in peri-infarct cortex of GFAP-TK mice (Figure 4M). Within GFAP-TK mice, peri-infarct IgG fluorescence, but not measures of vascular density (F_(1,6)_ = 4.67, p = 0.074, R^2^ = 0.44) or area fraction (F_(1,6)_ = 1.43, p = 0.277, R^2^ = 0.19), was significantly positively correlated with the number of caspase-3^+^ cells (F_(1,6)_ = 31.78, p = 0.0013, R^2^ = 0.84). Therefore, worsened blood-brain barrier dysfunction due to astrocyte loss is associated with continued cell death in the subacute period after stroke.

### Astrocyte ablation reduces basement membrane and mural cell coverage of vasculature in peri-infarct cortex

**Figure 5.**
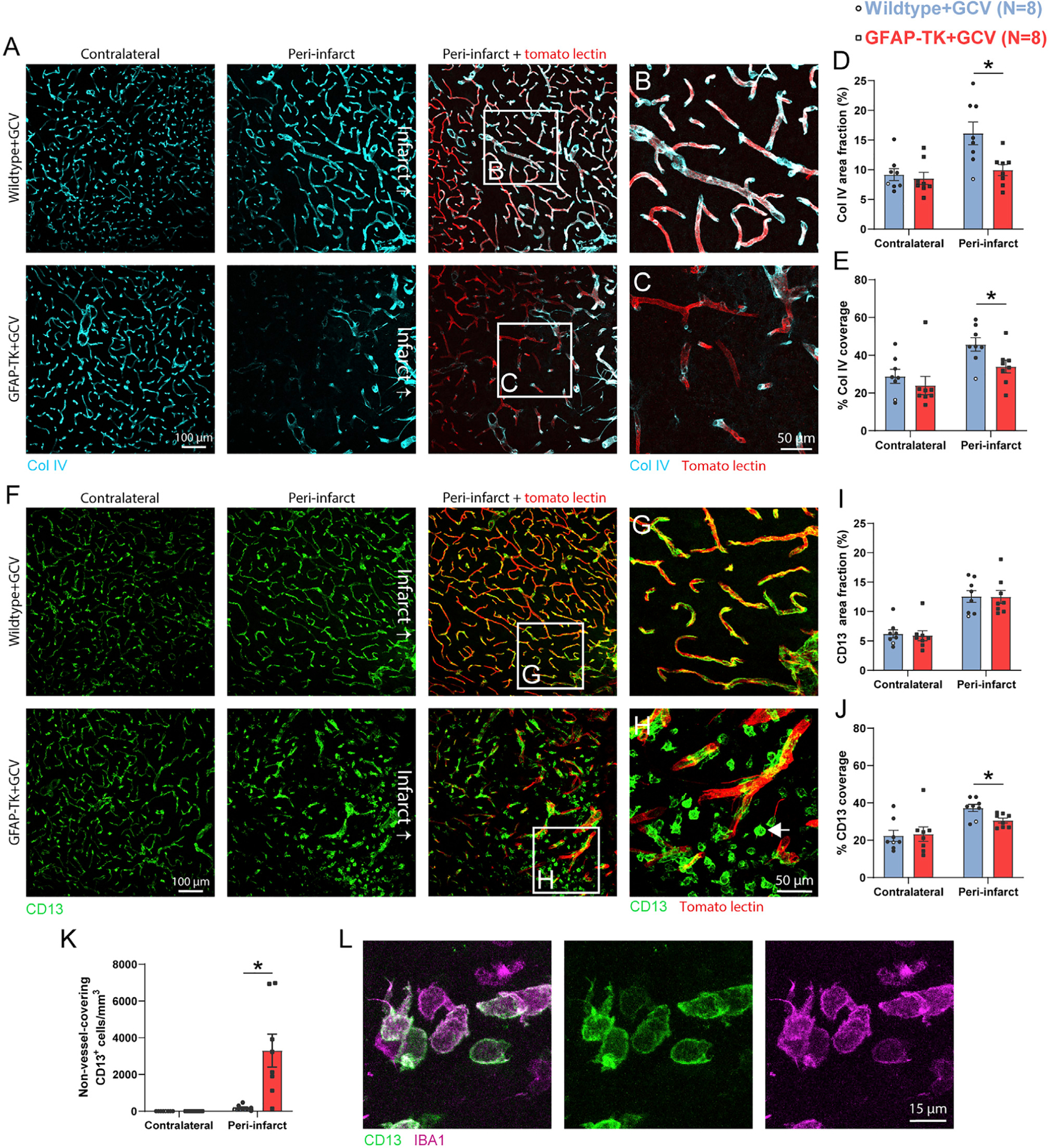
Astrocyte ablation reduces basement membrane and mural cell coverage of vasculature in peri-infarct cortex (A) Representative confocal images of collagen IV in peri-infarct and contralateral cortex 14 days post-stroke. (B and C) High-magnification images from regions indicated in (A). (D and E) Collagen IV area fraction (D) and vessel coverage (E) were significantly reduced in peri-infarct cortex of GFAP-TK+GCV mice (n = 8/group). *t_(14)_ ≥ 2.38, p ≤ 0.032, t tests. (F) Representative confocal images of pericytes (CD13^+^) in peri-infarct and contralateral cortex. (G and H) High-magnification images from regions indicated in (F). Large numbers of CD13^+^ cells not covering vessels were observed (arrow in H; quantified in K). (I) Peri-infarct CD13^+^ area fraction was not reduced by astrocyte ablation (t_(14)_ = 0.04, p = 0.967). (J) However, peri-infarct vascular coverage by pericytes was significantly reduced in GFAP-TK+GCV mice. *t(14) = 2.92, p = 0.011. (K) Quantification of non-vessel-covering CD13^+^ cells, which were IBA1^+^ (L), consistent with a myeloid cell phenotype. *t_(7.0)_ = 3.5, p = 0.010, Welch’s corrected t test. (L) Confocal images of non-vessel-associated CD13^+^ IBA1^+^ cells from peri-infarct cortex of a GFAP-TK+GCV mouse. Data are presented as mean ± SEM. Data points representing males are shown as filled symbols; data points representing females are shown as open symbols.

We next investigated the consequences of astrocyte ablation on two other components of the neurovascular unit involved in vascular repair and remodeling: basement membrane and pericytes. Extracellular matrix remodeling is critical for reorganization of vascular structure (Davis and Senger, 2005). Proper coverage of vessels by basement membrane, which is composed of extracellular matrix proteins, is associated with a mature vascular network and effective vascular repair, and likely contributes to blood-brain barrier properties (Thomsen et al., 2017; Xu et al., 2018). Astrocytes are a significant source of extracellular matrix (Davis and Senger, 2005; Xu et al., 2018), and stroke activates transcriptional programs relevant for extracellular matrix remodeling in astrocytes (Figure 1; Figure S2). We examined the effects of astrocyte ablation on collagen IV, the most abundant basement membrane component (Kang and Yao, 2020; Xu et al., 2018), in the remodeling vascular network in peri-infarct cortex. Immunostaining for collagen IV at day 14 showed that density and vessel coverage were not different in contralateral cortex. In contrast, there was reduced density of, and vessel coverage by, collagen IV in peri-infarct cortex of GFAP-TK mice (Figures 5A–5E). We confirmed this finding with similarly reduced vascular coverage by laminin, another main basement membrane component (Figure S5). We also assessed perineuronal nets, unique extracellular matrix structures that surround a subset of neurons and are implicated in modulating plasticity (Wang and Fawcett, 2012). We found a significant reduction in the number of *Wisteria floribunda* agglutinin (WFA)^+^ perineuronal nets due to stroke, but, in contrast to vascular-associated extracellular matrix, this reduction was not affected by astrocyte ablation (Figure S6). Therefore, ablation of reactive astrocytes disrupts vascular, but not neuronal, extracellular matrix reorganization. Sparse basement membrane coverage of peri-infarct vessels in GFAP-TK mice may reflect that the vascular network is immature, and could contribute to poor neovascularization and greater vascular permeability.

Pericytes are implicated in vascular proliferation and survival (Hellström et al., 2001). Moreover, extensive pericyte coverage of vasculature is associated with effective vascular repair after stroke, and pericyte coverage is reduced in several disease states (Rust et al., 2019b). We evaluated pericyte coverage of vessels by staining for CD13 (Figures 5F–5J). Despite no significant differences in the area fraction of CD13^+^ cells between groups in peri-infarct or contralateral cortex (Figure 5I), vessel coverage by pericytes was significantly reduced in peri-infarct cortex of mice in which reactive astrocytes were ablated (Figure 5J). The cause of this discrepancy between area fraction and vessel coverage was large numbers of CD13^+^ cells that were unattached to vessels in peri-infarct cortex of GFAP-TK mice (Figures 5G, 5H, and 5K). Non-vessel-covering CD13^+^ cells typically had ameboid morphology and were IBA1^+^, consistent with a myeloid cell phenotype (Figure 5L; Bush et al., 1999). Overall, impaired vascular repair due to astrocyte loss is associated with reduced pericyte coverage of vessels. It is possible that the sparse coverage by pericytes contributed to diminished neovascularization, heightened vascular regression, and vascular permeability. Moreover, sparse pericyte coverage could disrupt blood flow regulation, resulting in dissociations between neural activity and local blood flow (i.e., neurovascular uncoupling) (Hartmann et al., 2021; Kisler et al., 2020).

### Ablation of astrocytes in the otherwise intact brain does not affect vascular structure

**Figure 6.**
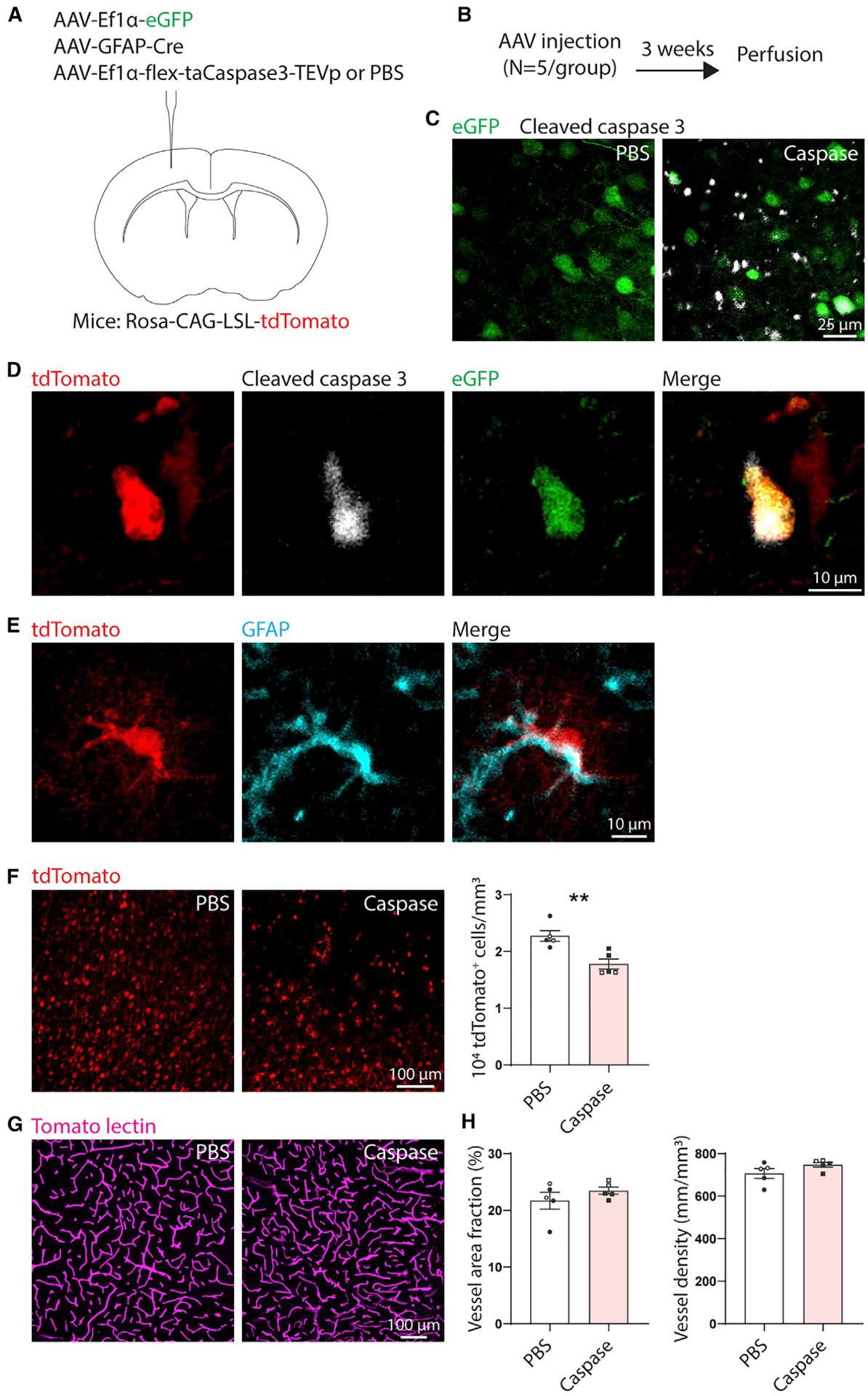
Genetic ablation of astrocytes in the otherwise intact brain does not affect vascular structure (A) AAV injection diagram. n = 5 Rosa-CAG-LSL-tdTomato mice per group (PBS or caspase). Mice in both groups were intracranially injected with AAV-EF1α-EGFP to label the injection site and AAV-GFAP-Cre to express Cre in astrocytes. Mice in the caspase group also received AAV-EF1α-flex-taCaspase3-TEVp to induce Cre-dependent expression of cleaved caspase-3, which causes apoptotic cell death. Control mice were injected with an equal volume of PBS instead of AAV-EF1α-flex-taCaspase3-TEVp. (B) Experimental timeline. (C) Confocal images showing expression of cleaved caspase-3 labeled by immunohistochemistry in mice injected with AAV-EF1α-flex-taCaspase3-TEVp (caspase), but not PBS. Images were taken at the injection site (EGFP label). (D) Example of a tdTomato^+^ cleaved caspase-3^+^EGFP^+^ cell (caspase group). Images are 5-μm maximum projections. (E) Example of a tdTomato^+^ GFAP^+^ cell, confirming Cre-mediated recombination in astrocytes. (F) The number of tdTomato^+^ cells was reduced in the caspase group, confirming ablation. Images are 6-μm maximum projections. **t_(8)_ = 3.82, p = 0.005, t test. (G) Representative maximum projection confocal images of tomato lectin-labeled vasculature at the injection site from caspase AAV- and PBS-injected animals. (H) Ablation of astrocytes did not affect vascular area fraction or density (t_(8)_ ≤ 1.6, p ≥ 0.146, t tests). Data are presented as mean ± SEM. Data points representing males are shown as filled symbols; data points representing females are shown as open symbols.

Our data indicate that astrocytes are crucial for post-stroke vascular remodeling. Impaired vascular remodeling is in part reflected in reduced vessel density in peri-infarct cortex of mice in which reactive astrocytes were ablated. Another possible explanation for this reduced vessel density is that astrocyte ablation itself (i.e., independent of injury) causes vessel loss. To test the possibility that astrocyte ablation alone causes vessel loss, we used a different genetic ablation approach in which we ablated astrocytes in the otherwise intact brain using Cre-dependent overexpression of cleaved caspase-3 (Yang et al., 2013). AAV-Ef1α-flex-taCaspase3-TEVp (caspase) or an equivalent volume of phosphate-buffered saline (PBS) was injected unilaterally into sensorimotor cortex along with AAV-GFAP-Cre to induce astrocytic Cre expression and AAV- Ef1α-EGFP to label the injection site (Figures 6A and 6B; n = 5 Rosa-CAG-LSL-tdTomato mice/group). AAV-Ef1α-flex-taCaspase3-TEVp induces Cre-dependent apoptotic cell death by 1 week post-infection (Yang et al., 2013). Animals were euthanized 3 weeks after AAV injection. We chose this time point to allow time for AAV-mediated gene expression followed by a delay similar to our post-stroke survival times. Caspase-injected mice had substantial cleaved caspase-3 expression and reduced numbers of astrocytes at the injection site (Figures 6C–6F). 61.7% ± 3.5% of tdTomato^+^ cells were S100β^+^, as counted from PBS-injected mice. Importantly, vascular structure was not different between caspase and PBS groups (Figures 6G and 6H). This finding is consistent with a study in adult GFAP-CreER^T2^:DTA mice in which no effects of astrocyte depletion on vascular structure or permeability were observed (Schreiner et al., 2015). These data support that focal astrocyte ablation does not cause vessel loss.

### Astrocyte ablation prolongs blood flow deficits and reduces vessel proliferation after stroke

**Figure 7.**
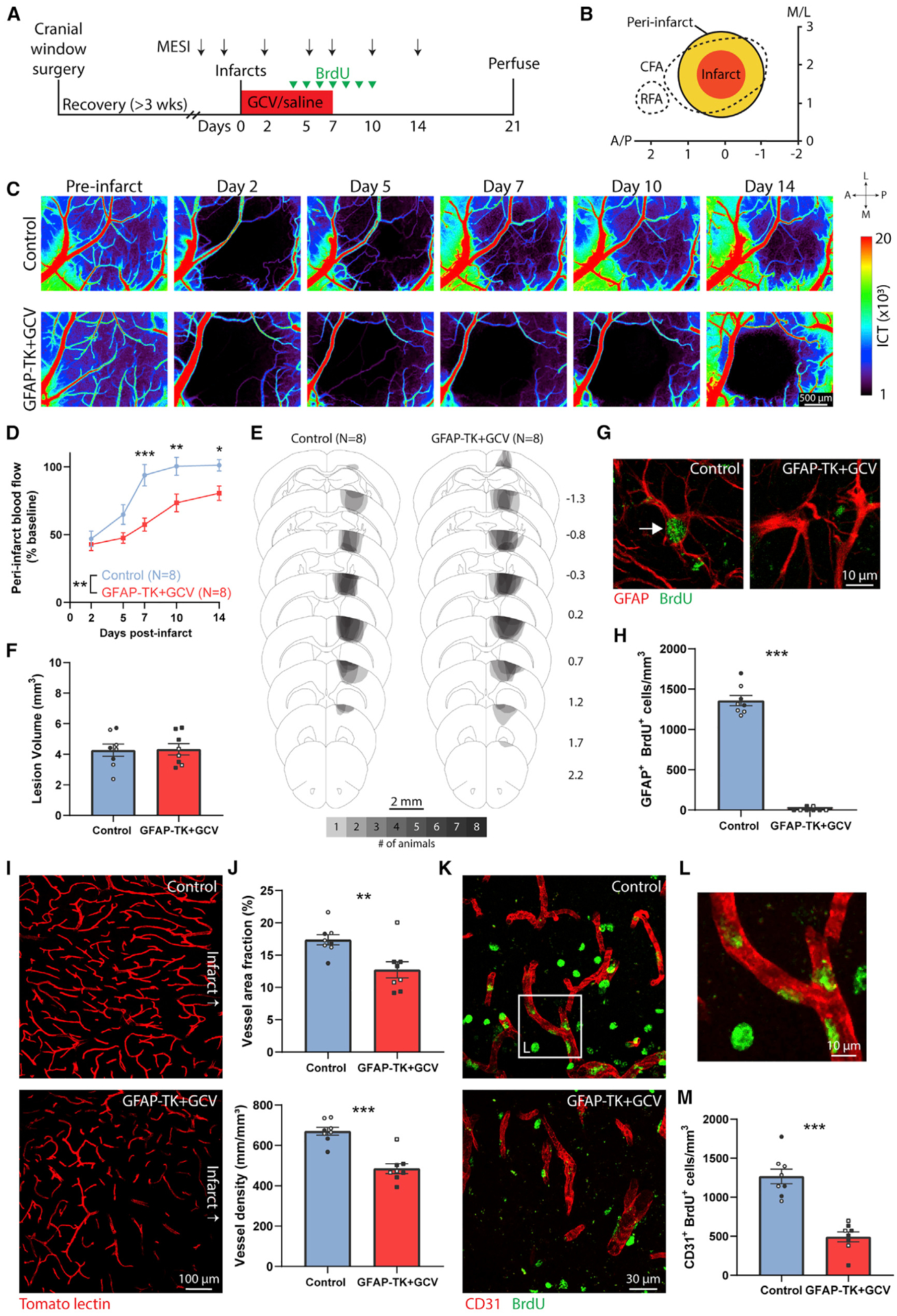
Astrocyte ablation limits restoration of blood flow and reduces vessel proliferation in peri-infarct cortex (A) Experimental timeline. n = 8 control mice (n = 5 wild-type+GCV, n = 3 GFAP-TK+saline), n = 8 GFAP-TK+GCV mice. (B) Schematic illustrating photothrombotic infarct placement relative to caudal (CFA) and rostral (RFA) forelimb areas in motor cortex. The infarct core was defined using the region of day 2 parenchymal blood flow with less than 20% of baseline parenchymal blood flow. Peri-infarct cortex was defined as extending 500 μm from the infarct border. Axes indicate mm relative to bregma. (C) Representative longitudinal MESI blood flow maps. (D) Peri-infarct blood flow deficits were prolonged in GFAP-TK mice given GCV. *p = 0.043, **p = 0.006, ***p = 0.0002, Holm-Sidak’s tests. (E) Schematic lesion reconstruction. Numbers on right indicate distance (mm) relative to bregma. (F) Lesion volume was not different between groups. t_(14)_ = 0.10, p = 0.919, t test. (G) Confirmation of ablation of proliferating astrocytes in peri-infarct cortex of GFAP-TK+GCV mice. Confocal images from peri-infarct cortex showing a GFAP^+^BrdU^+^ cell (arrow) in a control mouse and a GFAP^+^BrdU^−^ cell in a GFAP-TK+GCV mouse. (H) Quantification of peri-infarct GFAP^+^BrdU^+^ cells showing near complete ablation of proliferating astrocytes. ***U = 0, p = 0.0002, Mann-Whitney U test. (I) Representative confocal images of tomato lectin-labeled vessels in peri-infarct cortex. (J) Peri-infarct vessel area fraction and density were significantly reduced in mice with ablated astrocytes. **t_(14)_ = 3.14, p = 0.007; ***t_(14)_ = 5.93, p < 0.0001, t tests. (K) Representative confocal images (5-μm maximum projections) of CD31^+^ vasculature and BrdU in peri-infarct cortex. (L) High-magnification example of BrdU^+^CD31^+^ cells from the region indicated by the white box in (K). (M) The number of BrdU^+^CD31^+^ vessels was significantly reduced in GFAP-TK+GCV mice. ***t_(14)_ = 6.84, p < 0.0001, t test. Data are presented as mean ± SEM. When replicates are shown, data points representing males are shown as filled symbols; data points representing females are shown as open symbols.

Ischemic stroke causes a broad reduction in blood flow in peri-infarct regions that is associated with impaired neuronal function and repair (He et al., 2020; Mostany et al., 2010; Summers et al., 2017). Neovascularization facilitates reestablishment of normal blood flow, which enables behavioral recovery (Williamson et al., 2020). To assess whether the failure of vascular remodeling caused by astrocyte ablation affected the restoration of blood flow, we used multi-exposure speckle imaging (MESI) to track blood flow after stroke (n = 8 control mice [n = 5 wild-type+GCV, n = 3 GFAP-TK+saline], n = 8 GFAP-TK+GCV mice). MESI is an optical, contrast-free method suitable for longitudinal monitoring of cerebral blood flow (Clark et al., 2019a; He et al., 2020; Williamson et al., 2020). Mice were unilaterally implanted with chronic cranial windows over motor cortex and allowed at least 3 weeks to recover. Then, we repeatedly measured blood flow before and after photothrombotic infarcts out to 2 weeks post-stroke (Figure 7A). We assessed blood flow within ~500 μm of the infarct border (Figure 7B). Previous work has found substantial blood flow deficits in this region that predict behavioral deficits and normally recover by around 7 days post-infarct (Clark et al., 2019a; He et al., 2020; Williamson et al., 2020).

Blood flow measured in baseline (pre-infarct) sessions showed no differences between groups (inverse correlation time [ICT] values: control, 3,578 ± 206, GFAP-TK+GCV, 3,333 ± 151; t_(14)_ = 0.96, p = 0.354, t test). Stroke caused a reduction in peri-infarct blood flow to about 50% of baseline on day 2 in both groups (Figures 7C and 7D). Blood flow was restored to baseline levels in control mice by 7 days post-stroke. In contrast, mice with ablated astrocytes had prolonged deficits in blood flow (significant time by group interaction, F_(4, 56)_ = 4.0, p = 0.007, two-way repeated-measures ANOVA). Blood flow was significantly diminished in GFAP-TK+GCV mice relative to controls on days 7–14 post-stroke (Figure 7D). There were no differences between groups in lesion size or location (Figures 7E and 7F). The prolonged blood flow deficits due to astrocyte ablation may have contributed to delayed cell death, poor behavioral outcome, and hindered neural repair (Moeini et al., 2018; Mostany et al., 2010; Williamson et al., 2020).

To measure cell proliferation, we injected mice with BrdU once daily from days 4 to 10 post-stroke (Figure 7A). We used the number of GFAP^+^BrdU^+^ cells in peri-infarct cortex on day 21 post-stroke to confirm the near complete ablation of proliferating peri-infarct astrocytes in GFAP-TK+GCV mice, and large numbers in control mice (Figures 7G and 7H). Vascular remodeling was again significantly impaired in mice with ablated astrocytes, measured as reduced vessel density and area fraction in peri-infarct cortex (Figures 7I and 7J). Vascular plasticity occurs largely during the first 2 weeks post-stroke in this model (Williamson et al., 2020). To quantify vascular proliferation during the period of pronounced vascular plasticity, we counted the number of CD31^+^BrdU^+^ cells in peri-infarct cortex. The number of these cells was significantly reduced in GFAP-TK+GCV mice relative to controls (Figures 7K–7M). Glycocalyx coverage was again reduced in mice with ablated astrocytes (Figure S7). Altogether, our findings support that astrocytes facilitate vascular repair and remodeling after stroke.

## DISCUSSION

We investigated how reactive astrocytes contribute to vascular remodeling after stroke. We first showed that stroke induces transcriptional changes associated with vascular remodeling in reactive astrocytes. We demonstrated with *in vivo* imaging that astrocytes actively associate with new vessel segments after stroke. In the uninjured brain, focal astrocyte ablation does not affect vascular structure. However, we found that specific ablation of proliferating astrocytes in peri-infarct cortex caused a failure in the vascular remodeling response characterized by sparse vascularization; diminished coverage of vessels by basement membrane, pericytes, and glycocalyx; increased vascular permeability; diminished vascular proliferation; and prolonged blood flow deficits. In addition, astrocyte ablation caused prolonged peri-infarct cell death and worsened behavioral recovery. Our findings implicate reactive astrocytes as critical cellular mediators of vascular remodeling after stroke.

Stroke triggers a period of heightened vascular plasticity that rapidly restores blood flow to residual peri-infarct tissue and enables functional recovery (Williamson et al., 2020). While other factors may be involved, the failure of vascular remodeling due to astrocyte ablation is likely a key contributor to the poor behavioral outcome we observed. Neovascularization and subsequent reestablishment of blood flow likely restore normal neuronal function and create an environment suitable to support neural repair processes, including synaptic remodeling and cytogenesis (Brown et al., 2007; Mostany et al., 2010; Williamson et al., 2019). Vascular repair is also important for restoring blood-brain barrier properties (Munji et al., 2019; Rust et al., 2019c; Zlokovic, 2008). Although there are conflicting reports on the necessity of astrocytes for maintenance of the blood-brain barrier in the absence of injury (Heithoff et al., 2021; Kubotera et al., 2019; Schreiner et al., 2015), we found that astrocyte ablation exacerbated vascular permeability after stroke, which is consistent with work in other injury models (Bush et al., 1999; Faulkner et al., 2004; Vivinetto et al., 2020). This barrier dysfunction may be due to disrupted repair of several components of the neurovascular unit (e.g., glycocalyx, endothelial cells, basement membrane, and pericytes). Overall, our study highlights the importance of vascular repair and remodeling as a recovery mechanism.

Reactive astrogliosis has been associated with both limiting and enabling functional recovery. Our study supports pro-repair functions of reactive astrocytes after stroke. Some forms of injury and disease produce neurotoxic astrocytes that may exacerbate pathology (Guttenplan et al., 2020; Liddelow et al., 2017; Zamanian et al., 2012). In addition, reactive astrocytes have been thought to restrict neural repair, for example, by expression of growth inhibitory factors and formation of a scar (Burda and Sofroniew, 2014; Liuzzi and Lasek, 1987; Sofroniew and Vinters, 2010). However, recent work suggests that reactive astrocytes can facilitate axonal regrowth after CNS injury (Anderson et al., 2016). Moreover, substantive evidence from several traumatic injury models, as well as our data, has demonstrated that ablation of reactive astrocytes is associated with heightened inflammation and degeneration (Bush et al., 1999; Faulkner et al., 2004; Myer et al., 2006). These apparently conflicting functions of reactive astrocytes may be due to cellular heterogeneity and injury type-specific induction of different reactive phenotypes (Liddelow et al., 2017; Sofroniew, 2020; Zamanian et al., 2012). Therapies aimed at enhancing pro-reparative functions and reducing harmful functions in reactive astrocytes may benefit outcome.

We suspect that several mechanisms underlie the ability of reactive astrocytes to facilitate vascular repair and remodeling. Our analysis demonstrates that stroke upregulates an array of factors implicated in vascular sprouting and maturation, as well as extracellular matrix remodeling, within reactive astrocytes. While this analysis was based on gene expression data obtained from experiments that used a different mouse strain, age, and stroke model than our other experiments, given the complexity of interactions between cellular and structural components within the mature neurovascular unit, it follows that multiple signaling pathways orchestrating remodeling of multiple components would be required to enable effective repair. In support of this, we found that not only did astrocyte ablation impair neovascularization, but glycocalyx, basement membrane, and pericyte coverage of vessels were also markedly reduced. Thus, reactive astrocytes appear to instruct multiple aspects of vascular repair. Since the marked reductions in vessel density after astrocyte ablation likely cannot be explained solely due to new vessel formation (Williamson et al., 2020), it is probable that astrocytes protect and maintain the vascular network after stroke. In addition, it was recently shown that brain stab wound injury causes astrocyte endfeet, which cover blood vessels, to become enriched in mitochondria-endoplasmic reticulum contact sites (Gӧbel et al., 2020). Interfering with this enrichment diminished neovascularization and resulted in sparse vascularization around the lesion, suggesting that astrocytes provide metabolic support to remodeling vasculature. Our finding that newly formed vessels were contacted by astrocytes corroborates the importance of astrocytic contact with remodeling vessels. Finally, delayed upregulation of HMGB1 in peri-infarct reactive astrocytes was reported to attract circulating endothelial progenitor cells, which may contribute to vascular remodeling (Hayakawa et al., 2010, 2012). Altogether, these data support multiple mechanisms by which reactive astrocytes can promote vascular repair and remodeling.

Stroke incidence is highest in the aged population (Feigin et al., 2014). Normal aging causes astrocytic gene expression signatures to resemble a reactive state (Androvic et al., 2020; Boisvert et al., 2018; Clarke et al., 2018), suggesting that aged astrocytes may be excessively primed to become reactive in response to insults. Indeed, peri-lesional astrocytes adopt reactive morphology sooner after injury in aged relative to younger animals (Popa-Wagner et al., 2007). Stroke causes a short period of vascular plasticity that is coincident with transient transcriptional changes in remodeling endothelial cells (Rust et al., 2019b; Williamson et al., 2020). However, age diminishes the vascular remodeling response and associated gene expression changes (Buga et al., 2014). Given these age-related changes in astrocytic and vascular responses to stroke, future studies should address how age modifies astrocyte-vasculature interactions during neovascularization.

We demonstrated a crucial role for reactive astrocytes in protecting pre-existing vessels and facilitating vascular repair and remodeling after stroke. Given that post-stroke vascular remodeling contributes to behavioral recovery, dissection of the cell types and pathways that control it could be of therapeutic value. Although some functions of reactive astrocytes, such as scar formation and expression of growth inhibitory factors, have been viewed as a barrier to repair and recovery, our study illustrates that reactive astrocytes can have active roles in promoting at least some neural repair processes that enable functional recovery.

## STAR★METHODS

### RESOURCE AVAILABILITY

#### Lead contact

Further information and requests for resources and reagents should be directed to and will be fulfilled by the Lead Contact, Michael Williamson (mrwillia@utexas.edu).

#### Materials availability

This study did not generate new unique reagents.

#### Data and code availability

Source dataset related to Figure 1 is available from Gene Expression Omnibus GSE35338.

### EXPERIMENTAL MODEL AND SUBJECT DETAILS

Adult transgenic and wild-type littermate mice on a C57BL/6 background were used (2.5–6 months old, 22–33 g). Transgenic strains were Rosa-CAG-LSL-tdTomato (Ai14, Cre-dependent tdTomato, JAX #007914) and GFAP-TK (JAX #005698) (Garcia et al., 2004; Swan et al., 2014). Both male and female mice were used. When replicates are shown, datapoints representing males are shown as filled symbols; datapoints representing females are shown as open symbols. Animals were housed 2–5 per cage with free access to food and water. When not dependent on genotype, animals were randomized to groups. Animal use was in accordance with a protocol approved by the Institutional Animal Care and Use Committee at the University of Texas at Austin. Experimenters were blinded to group allocation. Experiments consisted of 1–4 cohorts of animals, with each cohort consisting of animals from both groups. Sample sizes were based on published (Clark et al., 2019a; Rust et al., 2019b; Williamson et al., 2020) and unpublished studies using similar methods. No formal *a priori* statistical estimation was used to determine sample sizes.

### METHOD DETAILS

#### Astrocyte transcriptome analysis

Microarray gene expression data from astrocytes after middle cerebral artery occlusion or sham procedures was accessed from Gene Expression Omnibus GSE35338 (Zamanian et al., 2012). Data across three survival times was pooled to yield N = 11 stroke and N = 10 sham samples during the first week post-surgery (post-surgery day 1: 5 stroke, 4 sham; day 3: 3 stroke, 3 sham; day 7: 3 stroke, 3 sham). Gene expression was compared between groups using GEO2R. For gene ontology enrichment analysis, the target set of differentially expressed genes (DEGs) was defined as genes with log2 fold change > 1 or < −1 and FDR adjusted p < 0.05 in post-stroke samples relative to sham samples. Gene ontology term enrichment was evaluated with Metascape (Zhou et al., 2019) and GOrilla (Eden et al., 2009) using all upregulated or downregulated DEGs as the target set. All genes in the dataset were used as the background set for GOrilla analysis. Conclusions from these analyses were similar with a stricter definition of DEGs (log2 fold change > 2 or < −2 and adjusted p < 0.05).

#### Cranial windows

Cranial windows were created over forelimb motor cortex as described (Clark et al., 2019a; Williamson et al., 2020). Mice were anesthetized with isoflurane (3% induction, 1%–2% maintenance) in oxygen and placed in a stereotaxic frame. 2 mg/kg dexamethasone and 2.5 mg/kg carprofen were given s.c.. A 4.5 mm circular craniotomy centered 1.5 mm lateral to Bregma was made with a dental drill. The dura was left intact. Saline-soaked Gelfoam (Pfizer) was used to control bleeding. A 4 mm #1 cover glass (Warner Instruments) was gently positioned over the craniotomy and secured in place with cyanoacrylate and dental cement. 0.05 mg/kg buprenorphine (s.c.) was given after surgery for pain management. 2.5 mg/kg carprofen was given daily for the following 7 days to minimize inflammation-induced deterioration of optical clarity.

#### Virus injections

Cortical astrocytes were sparsely labeled in two Rosa-CAG-LSL-tdTomato mice by stereotactic intracranial microinjections of AAV8-GFAP-mCherry-Cre virus (UNC Vector Core) immediately after the craniotomy during cranial window implantation. We deliberately chose a method that would yield sparse labeling in order to ease visualization of fine processes since dense labeling can make this difficult. AAV itself induces transient and mild astrogliosis that can induce Gfap promoter-driven transgene expression by transiently upregulating GFAP (Ortinski et al., 2010). Virus was injected (23 or 46 μL per site) with a Drummond Nanoject II microinjector through a pulled pipette at 10–12 sites throughout sensorimotor cortex. Injections were made at a depth of 100–400 μm from the brain surface. Virus was injected 23 nL at a time, once per minute. The pipette was left in place for an additional 1–2 minutes after the final injection at each site.

AAV5-EF1α-flex-taCaspase3-TEVp (UNC Vector Core) (Yang et al., 2013) or an equivalent volume of PBS (N = 5 Rosa-CAG-LSL-tdTomato mice/group), AAV5-GFAP-Cre (Addgene), and AAV5-EF1α-eGFP (Addgene) were unilaterally microinjected in a 2:2:1 ratio. Injections were made throughout sensorimotor cortex at four locations in the XY plane spaced approximately 500 μm. At each XY location, injections of 92 nL were made at two depths (300–400 and 700–800 μm) below the pial surface. Virus was injected 23 nL at a time, once per minute. The pipette was left in place for an additional minute after the final injection at each site.

#### Stroke model

Photothrombotic infarcts were created in forelimb motor cortex. Mice were anesthetized with isoflurane (3% induction, 1.5% maintenance) in oxygen and headfixed. Body temperature was maintained with a heated pad. For infarcts induced through cranial windows, leptomeningeal vessels were visualized with real-time laser speckle contrast imaging. A penetrating arteriole supplying the forelimb motor region (Tennant et al., 2011) was illuminated with a 20 mW 532 nm laser (~300 μm spot size) for 15 minutes beginning 3 minutes after 0.2 mL of 15 mg/kg rose bengal was given i.p. (Clark et al., 2019a, 2019b). Successful occlusion was monitored and confirmed by continuous speckle imaging. For infarcts induced through the intact skull, a midline scalp incision was made and the skull was cleaned. The skull was illuminated with white light from a surgical lamp (Schott KL 200, maximum power, 15 minutes) through a 3 mm aperture centered 2 mm lateral from Bregma beginning 3 minutes after i.p. injection of 0.15 mL of 15 mg/mL rose Bengal (Lee et al., 2004).

#### In vivo 2-photon imaging

Mice were anesthetized with isoflurane (3% induction, ~1.5% maintenance) in oxygen and headfixed. Body temperature was maintained with a heated pad. 0.1 mL of 5% (w/v) 70 kDa fluorescein isothiocyanate (FITC)-dextran (Sigma, 46945) was injected retro-orbitally to label vasculature. Two-photon imaging was performed using a mode-locked femtosecond multi-photon microscope (MaiTai, Spectra Physics) tuned to 730 nm. Image stacks were acquired with 512 × 512 pixel resolution and 2.0 μm z step size using a water-immersion 20 × /1.0NA (Olympus) objective. Fluorescent emission from RFP and FITC-dextran was detected by PMTs after passing through a 595 ± 35 nm or 525 ± 25 nm bandpass filter, respectively.

#### Bromodeoxyuridine administration

100 mg/kg of BrdU (Sigma; 10 mg/mL in saline) was injected i.p. once or twice per day from days 4–10 post-stroke.

#### Ganciclovir administration

100 mg/kg/day of ganciclovir (Roche, Cytovene) in saline was delivered for 7 days via osmotic pumps (Alzet, 1007D) (Anderson et al., 2016; Bush et al., 1999). Pumps were implanted into a subcutaneous pocket on the back immediately before photothrombosis.

#### Behavioral testing

All behavioral tests included pre-infarct test sessions to establish baseline performance. Experimenters were blind to group allocation during testing and offline rating.

The cylinder test was used to examine forelimb use asymmetry during vertical exploration, which involves animals rearing to an upright position and using one or both forelimbs for support against the cylinder wall (Schallert et al., 2000). Mice were placed in a 10 cm diameter 15 cm tall plexiglass cylinder on a clear plexiglass surface and recorded form below. Animals were recorded for at least 2 minutes and until at least 10 rears were observed. Videos were rated offline. Only rears in which both forelimbs could be clearly seen were rated. The median number of rears rated per session was 14. During each rear, weight-bearing forelimb use was rated as using both forelimbs, the left forelimb, or the right forelimb (Schallert et al., 2000). A forelimb was defined as not used if it was not used in a weight-bearing manner for more than one third of the time during a given rear. The % impaired forelimb use was calculated as: %(impaired forelimb use + ½ both forelimb use)/total forelimb use.

The grid walking test was used to assess motor deficits during locomotion (Baskin et al., 2003). Mice were placed on a 45 × 35 cm wire grid with 1 cm openings. Each animal was recorded from below for at least 2 minutes. Videos were rated offline. Only steps in which the paw was clearly visible were counted. Stepping faults in the impaired forelimb were counted relative to the total number of steps counted (100 steps per trial). Faults were defined as steps where the paw missed the wire grid and went through a hole or when the wrist or forearm, rather than the paw, were used for support.

#### Multi-exposure speckle imaging of blood flow

MESI was done as previously described (Clark et al., 2019a; Williamson et al., 2020). MESI provides a label-free, quantitative measure of micro- and macrovascular perfusion. Mice were anesthetized with isoflurane (3% induction, 1.25% maintenance) in oxygen and headfixed. The oxygen flow rate and concentration of isoflurane were kept the same for each imaging session to control for effects of anesthesia on blood flow. Body temperature was maintained with a heated pad. The craniotomy was illuminated with a 685 nm laser. Backscattered light was collected by a camera (Basler) over 15 exposure durations ranging from 0.05 to 80 ms. The amplitude of the illumination was controlled by an acousto-optic modulator. Raw images were processed with MATLAB to produce inverse correlation time (ICT) images, where ICT is proportional to blood flow (Clark et al., 2019a). Each imaging session lasted < 10 minutes. Two pre-infarct images were collected on different days at least 3 weeks after cranial windows were implanted. Post-infarct images were collected on days 2, 5, 7, 10, and 14.

ICT maps were analyzed with ImageJ (Williamson et al., 2020). Blood flow was measured in 8–10 randomly sampled parenchymal regions in each of the two baseline images. Mean ICT was averaged across these regions and then across baseline images to yield mean baseline parenchymal blood flow. Peri-infarct blood flow was measured by averaging the mean ICT values from 8–10 randomly sampled parenchymal regions within ~500 μm of the infarct border in all post-stroke images (Williamson et al., 2020). The same regions were measured in each post-infarct image. Blood flow is expressed as a percentage of mean baseline blood flow.

#### Histology

Animals were overdosed with Euthasol and transcardially perfused with 0.1M phosphate buffer followed by 4% paraformaldehyde in phosphate buffer. Brains were removed and post fixed overnight at 4°C in 4% paraformaldehyde. Brains were coronally sectioned at 35 μm with a vibratome (VT1000S, Leica). Sections were stored in cryoprotectant at −20°C.

To label vasculature, animals were briefly anesthetized with isoflurane and given a retro-orbital injection of Dylight 649-conjugated tomato lectin (100 μL, Vector Labs, DL-1178). Animals were allowed to recover for 5 minutes before perfusion. At least 4 sections spaced ≥ 175 μm were mounted immediately after sectioning for imaging vasculature.

A set of every fifth section stained with toluidine blue was used for lesion reconstruction and to determine lesion volume. Lesions were reconstructed onto schematic coronal sections as described (Kim et al., 2018). Cortical volume in each hemisphere was calculated using Cavalieri’s method as the sum of area of cortex × distance between section planes. Lesion volume was determined as the volume of intact cortex – volume of injured cortex (Kim et al., 2018).

For immunohistochemistry, sections were first washed in PBS. When staining for collagen IV or laminin, sections were treated with 1 mg/mL pepsin in 0.2 N HCl for 10 minutes at 37°C. When staining for BrdU, sections were incubated in 2 N HCl for 30 minutes followed by 0.1 M boric acid for 10 minutes. Sections were then blocked in 10% donkey serum in PBS with 0.25% Triton, and then incubated overnight at room temperature with primary antibodies or biotinylated WFA (1:500, Vector Labs, B-1355). Primary antibodies were: goat anti-Aldh1l1 (1:25, Rockland, 600–101-HB6), rabbit anti-aquaporin 4 (1:2000, Millipore, AB3594), mouse anti-BrdU (1:1000, Invitrogen, MA3–071), rabbit anti-BrdU (1:1000, Abcam, ab152095), rat anti-BrdU (1:1000, Abcam, ab6326), goat anti-CD13 (1:50, R&D Systems, AF2335), rat anti-CD31 (1:50, BD PharMingen, 550274), rabbit anti-cleaved caspase 3 (1:250 or 1:400, Cell Signaling, #9661), rabbit anti-collagen IV (1:500, Abcam, ab6586), rabbit anti-GFAP (1:1000, Dako, Z0334), rabbit anti-IBA1 (1:1000, Wako, 019–19741), rabbit anti-laminin (1:200, Abcam, ab11575), goat anti-HSV thymidine kinase (1:1000, Santa Cruz Biotechnology, sc-28038), and rabbit anti-S100β (1:1000, Abcam, ab52642). Sections were washed and then incubated for 1 hour with Alexa Fluor 405-, 488-, 594, or 647-conjugated species-appropriate secondary antibodies raised in donkey (1:500, Jackson ImmunoResearch) or Cy3-conjugated streptavidin (1:250, ThermoFisher, SA1010) in blocking solution. Alexa Fluor 488-conjugated donkey anti-mouse IgG (1:500) was used to stain for IgG. Sections were then washed, mounted, and coverslipped with Fluoromount G with or without DAPI (ThermoFisher).

Fluorescent images were collected with a Leica TCS SP5 confocal microscope using a 20 × /0.7NA or 40 × /1.0NA objective. Image stacks were collected with 2–6 frame averaging and a 1–2 μm step size between optical planes. Imaging parameters were consistent between samples for each label within each experiment.

Image analysis was done with FIJI. Images from 3–4 sections per mouse were analyzed for each region of interest. For area fraction calculation, maximum intensity projection images were automatically and uniformly processed: background was subtracted, images were thresholded and denoised, and the Analyze Particles tool was used to measure area fraction. Vessel length was determined using the Skeletonize and Analyze Skeleton tools on binarized vessel images (Williamson et al., 2020). Line profiles measuring lectin fluorescence were used to determine glycocalyx coverage. At least 60 line profiles from at least 3 images per mouse were measured perpendicular to discrete capillary segments in maximum projection 8-bit tomato lectin images. The mean lectin fluorescence within each vessel was calculated as the average value of pixels greater than half of the maximum pixel value across the line scan, approximating full-width at half-maximum segmentation (Kutuzov et al., 2018; Summers et al., 2017). To calculate lectin fluorescence or collagen, laminin, or pericyte vessel coverage in all vessels, a binarized projection of tomato lectin-labeled vasculature was used as a mask. Lectin fluorescence was calculated as the mean pixel intensity within the masked region. The collagen IV^+^, laminin^+^, or CD13^+^ area fraction within the masked region was defined as the percent coverage. For cell density quantification, cells were counted from confocal image stacks using the optical disector method and an unbiased counting frame. Cell density was calculated as the number of cells / (frame area × section thickness).

### QUANTIFICATION AND STATISTICAL ANALYSIS

Data are expressed as mean ± SEM. Additionally, measurements from individual animals are shown on plots as uncolored datapoints where possible. Data were analyzed with GraphPad Prism version 8.4. Independent samples were compared with two-tailed t tests. Variance was assessed with F tests, and Welch’s corrected t tests were used in cases where variance was significantly different. Mann Whitney U tests were used when data were not normally distributed. Two-way ANOVAs and linear regressions were used as noted in the text. Post hoc Holm-Sidak’s tests were used following significant ANOVA. Alpha was set at p < 0.05. Details on the statistical tests used for each experiment are located in the Results and figure legends.

## Supplementary Material

1

2

## Figures and Tables

**Table:** KEY RESOURCES TABLE

REAGENT or RESOURCE	SOURCE	IDENTIFIER
Antibodies		
Goat polyclonal anti-Aldh1l1	Rockland	600-101-HB6
Rabbit polyclonal anti-aquaporin 4	Millipore	AB3594; RRID:AB_91530
Mouse monoclonal anti-BrdU	Invitrogen	MA3-071; RRID:AB_10986341
Rabbit polyclonal anti-BrdU	Abcam	ab152095; RRID:AB_2813902
Rat monoclonal anti-BrdU Abcam		ab6326; RRID:AB_305426
Goat polyclonal anti-CD13	R&D Systems	AF2335; RRID:AB_2227288
Rat monoclonal anti-mouse CD31	BD PharMingen	550274; RRID:AB_393571
Rabbit polyclonal anti-cleaved caspase 3	Cell Signaling	#9661; RRID:AB_2341188
Rabbit polyclonal anti-collagen IV	Abcam	Ab6586; RRID:AB_305584
Rabbit polyclonal anti-GFAP	Dako	Z0334; RRID:AB_10013382
Rabbit polyclonal anti-IBA1	Wako	019–19741; RRID:AB_839504
Rabbit polyclonal anti-laminin	Abcam	Ab11575; RRID:AB_298179
Goat polyclonal anti-HSV thymidine kinase	Santa Cruz Biotech	Sc-28038; RRID:AB_675911
Rabbit monoclonal anti-S100b	Abcam	Ab52642; RRID:AB_882426
Bacterial and virus strains		
AAV8-GFAP-mCherry-Cre	UNC Vector Core	N/A
AAV5-EF1α-flex-taCaspase3-TEVp	UNC Vector Core	N/A
AAV5-GFAP-Cre	Addgene	Cat# 105550-AAV5
AAV5-EF1α-eGFP	Addgene	Cat# 105547-AAV5
Chemicals, Peptides, and Recombinant Proteins		
Rose Bengal	Sigma	Cat# 330000
Bromodeoxyuridine	Sigma	Cat# B5002
FITC-dextran	Sigma	Cat# 46945
Ganciclovir	Roche	N/A
Deposited data		
Expression data from reactive astrocytes acutely purified from young adult mouse brains	Zamanian et al., 2012	GSE35338
Experimental models: Organisms/strains		
Mouse: Ai14: B6.Cg-Gt(ROSA)26Sortm14 (CAG-tdTomato)Hze/J	The Jackson Laboratory	JAX #007914; RRID:IMSR_JAX:007914
Mouse: GFAP-TK: B6.Cg-Tg(Gfap-TK)7.1Mvs/J	The Jackson Laboratory	JAX #005698; RRID:IMSR_JAX:005698
Software and Algorithms		
FIJI	NIH	https://imagej.net/Fiji
GraphPad Prism	GraphPad Software	V8.4
Other		
Biotinylated Wisteria Floribunda Lectin	Vector Labs	B-1355; RRID:AB_2336874
Dylight 649-conjugated tomato lectin	Vector Labs	DL-1178

## Highlights

- Reactive astrocytes upregulate angiogenesis-related genes after stroke
- Astrocytes interact with new vessels in peri-infarct cortex
- Ablation of reactive astrocytes disrupts vascular repair and remodeling
- Ablation of reactive astrocytes worsens motor recovery
