# Supplementary material for: Reactive astrocytes facilitate vascular repair and remodeling after stroke
Source: Cell Rep. Author manuscript; Available in PMC 2021 May 24. (PMC8142687; doi:10.1016/j.celrep.2021.109048)
Supplement: 1 [file NIHMS1698680-supplement-1.pdf]

Cell Reports, Volume *35*

Supplemental information

Reactive astrocytes facilitate vascular repair and remodeling after stroke

Michael R. Williamson, Cathleen Joy A. Fuertes, Andrew K. Dunn, Michael R. Drew, and Theresa A. Jones

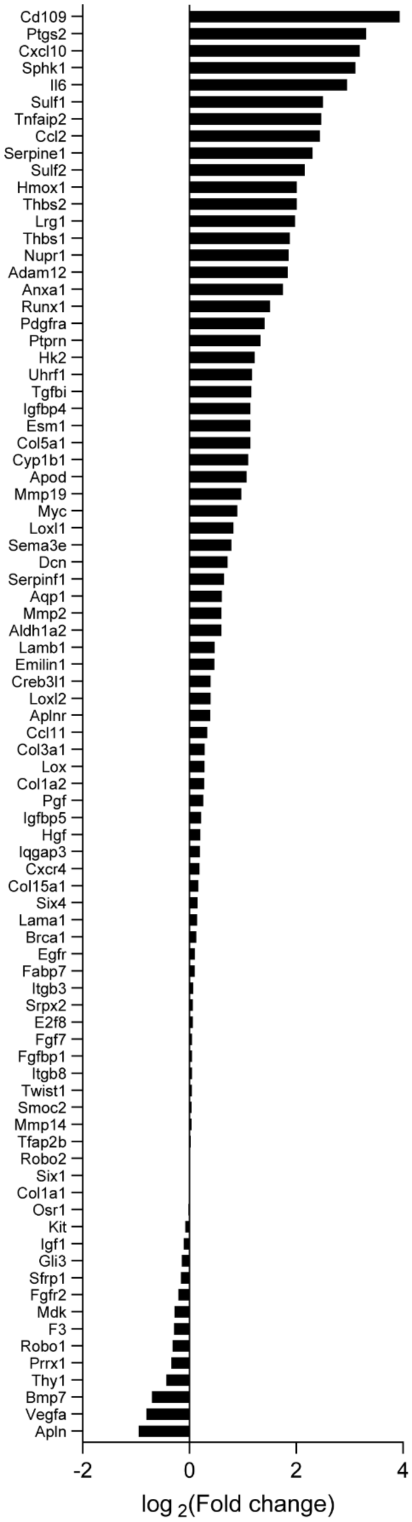

Figure S1. Expression of angiogenesis-related genes in reactive astrocytes after stroke. Related to Figure 1.

Expression of angiogenesis-related genes (GO:0001568) in astrocytes after stroke. Data are expressed as log2(fold change) in astrocytes from stroke vs. sham animals.

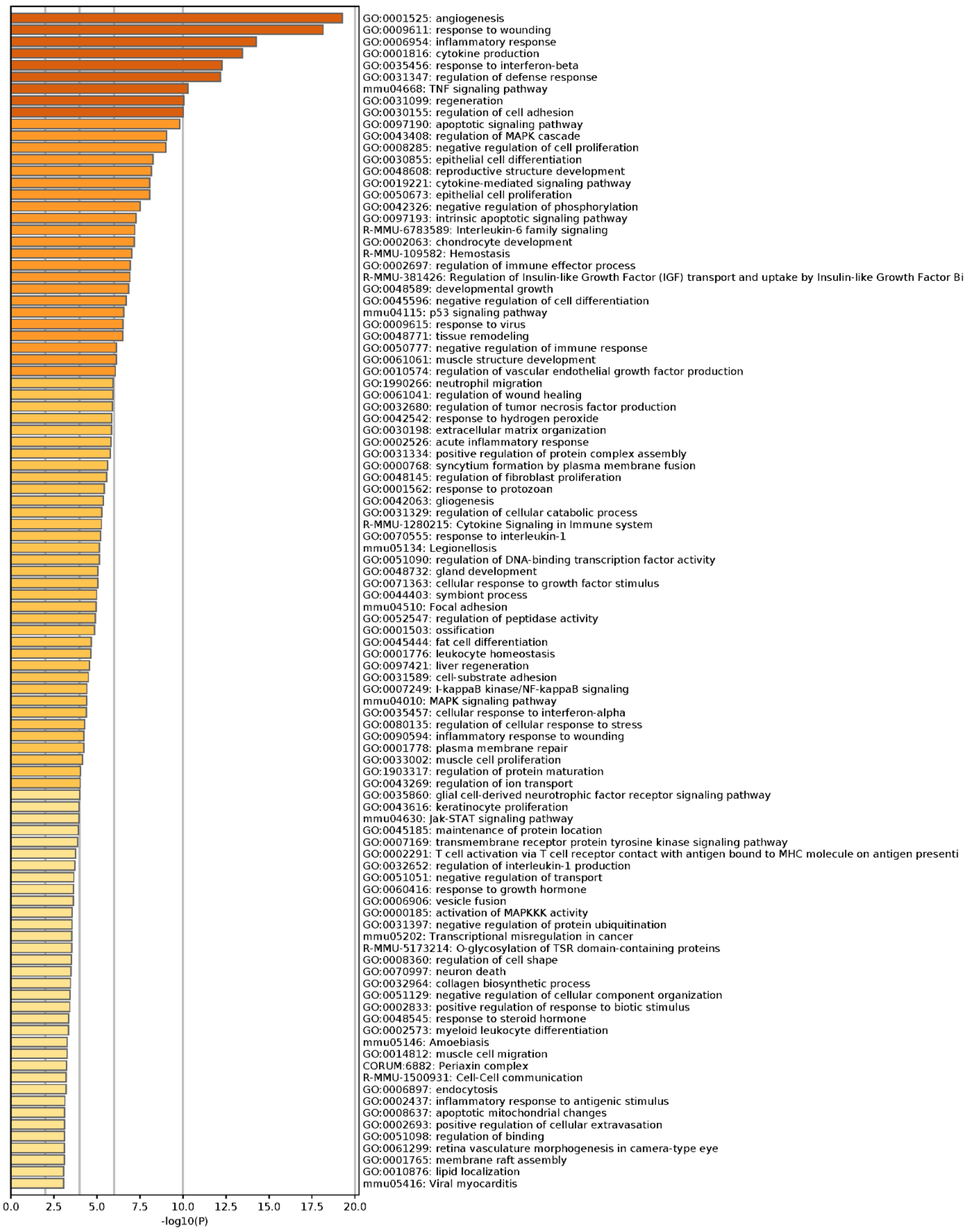

Figure S2. Extended list of enriched gene ontology terms in reactive astrocytes after stroke. Related to Figure 1.

Top 100 enriched gene ontology terms for upregulated genes in reactive astrocytes after stroke. Terms are sorted and color-coded by P value.

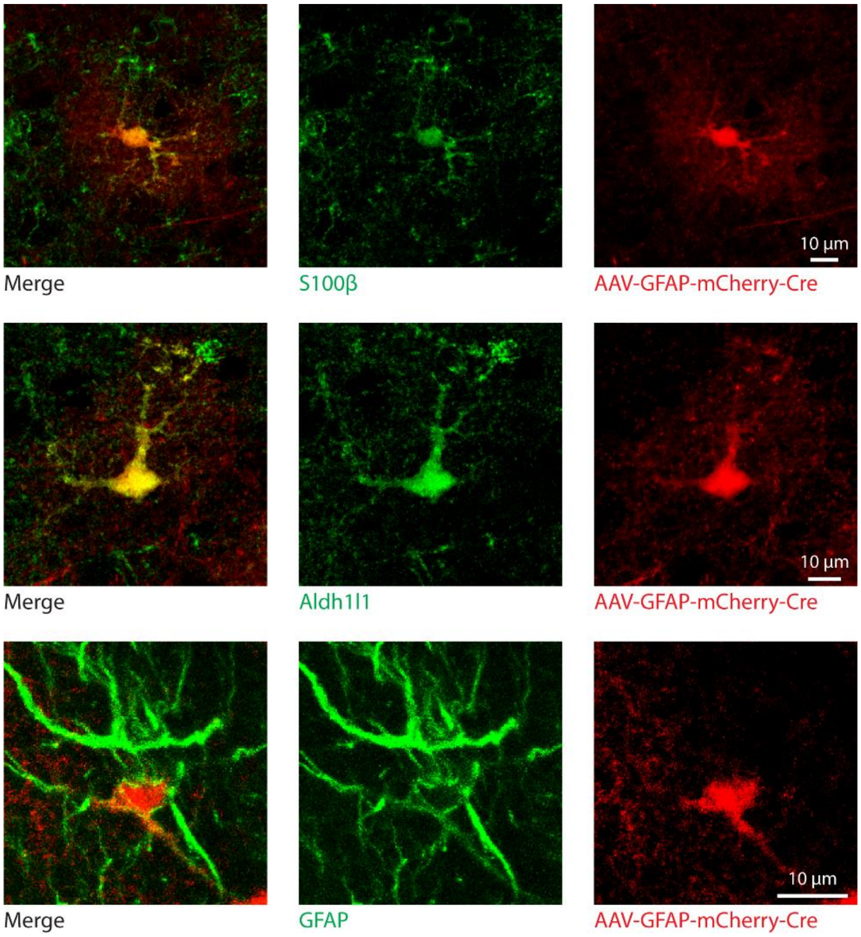

Figure S3. AAV-mediated labeling of astrocytes. Related to Figure 2.

Confocal images of AAV-GFAP-mCherry-Cre-labeled cells expressing astrocyte markers Aldh1l1, S100β, and GFAP.

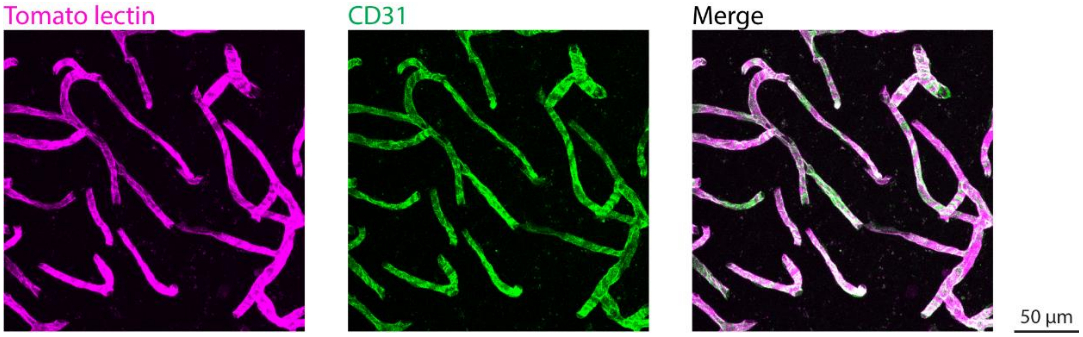

Figure S4. Validation of endothelial cell labeling with tomato lectin. Related to Figure 4.

Confocal images of tomato lectin+ and CD31+ endothelial cells in cortex. Note that lectin labeling is restricted to CD31+ endothelial cells.

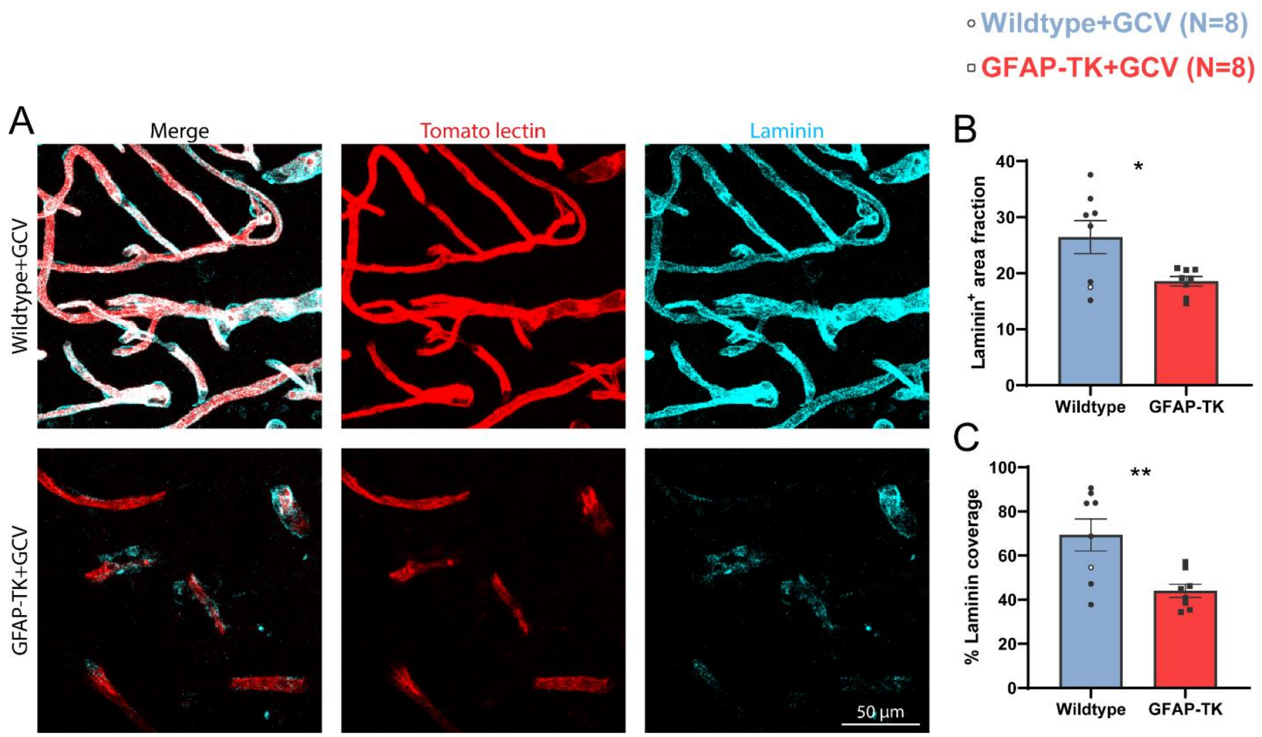

Figure S5. Reduced peri-infarct vessel coverage by laminin in GFAP-TK+GCV mice. Related to Figure 5.

A) Confocal images of laminin and tomato lectin-labeled blood vessels in peri-infarct cortex. Quantification of laminin area fraction (B) and vascular coverage (C) in peri-infarct cortex. *$t(8.2) = 2.6$, $P = 0.0318$, **$t(9.3) = 3.2$, $P = 0.0097$, Welch's corrected t tests. Datapoints representing males are shown as filled symbols; datapoints representing females are shown as open symbols.

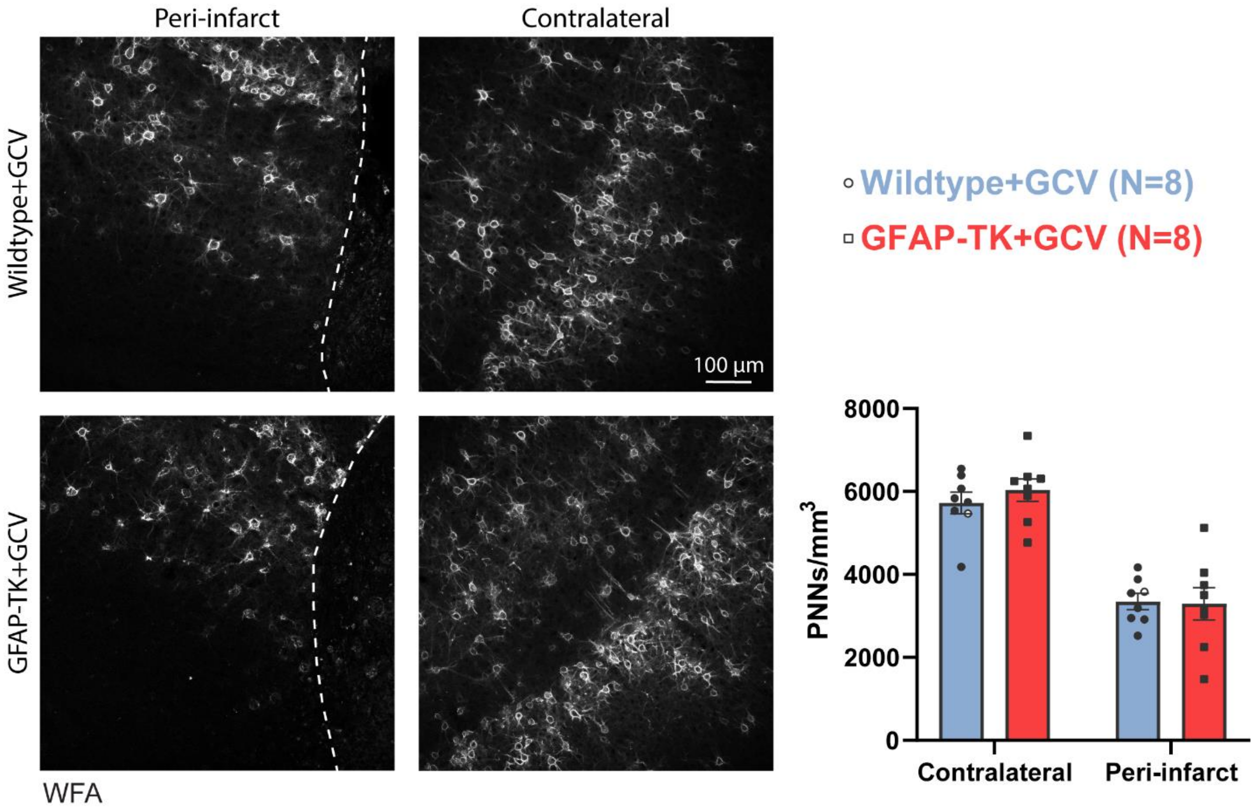

Figure S6. Astrocyte ablation did not affect perineuronal nets. Related to Figure 5.

Representative images of WFA-labeled perineuronal nets in peri-infarct and homotopic contralateral cortex. Dashed lines represent the lesion border. The number of perineuronal nets was significantly reduced in peri-infarct cortex in both groups relative to contralateral cortex ($t(14) \leq 7.36$, $P < 0.0001$, t tests between regions for each group). The number of perineuronal nets was not different between groups in either region ($t(14) \leq 0{,}83$, $P \geq 0.420$, t tests between groups for each region). Datapoints representing males are shown as filled symbols; datapoints representing females are shown as open symbols.

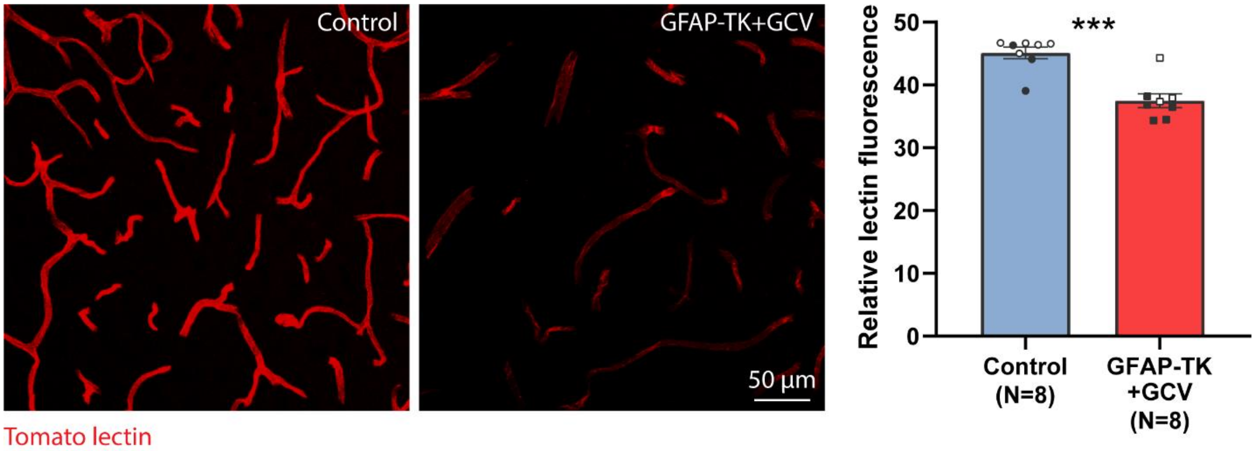

Figure S7. Astrocyte ablation reduces endothelial glycocalyx content at 21 days post-stroke. Related to Figure 7.

Confocal images of tomato lectin (left) and quantification of lectin fluorescence within peri-infarct vessels (right) to measure glycocalyx content. Data correspond to the experiment shown in Figure 7. Datapoints representing males are shown as filled symbols; datapoints representing females are shown as open symbols. ***$t(14) = 5.3$, $P < 0.0001$.
